# Cross-Platform Toxicogenomics for the Prediction of Non-Genotoxic Hepatocarcinogenesis in Rat

**DOI:** 10.1371/journal.pone.0097640

**Published:** 2014-05-15

**Authors:** Michael Römer, Johannes Eichner, Ute Metzger, Markus F. Templin, Simon Plummer, Heidrun Ellinger-Ziegelbauer, Andreas Zell

**Affiliations:** 1 Center of Bioinformatics Tuebingen (ZBIT), University of Tuebingen, Tübingen, Germany; 2 Natural and Medical Sciences Institute at the University of Tübingen, Reutlingen, Germany; 3 CXR Biosciences, James Lindsay Place, Dundee Technopole, Dundee, Scotland, United Kingdom; 4 Bayer Pharma AG, Wuppertal, Germany; CSIR-Institute of Microbial Technology, India

## Abstract

In the area of omics profiling in toxicology, i.e. toxicogenomics, characteristic molecular profiles have previously been incorporated into prediction models for early assessment of a carcinogenic potential and mechanism-based classification of compounds. Traditionally, the biomarker signatures used for model construction were derived from individual high-throughput techniques, such as microarrays designed for monitoring global mRNA expression. In this study, we built predictive models by integrating omics data across complementary microarray platforms and introduced new concepts for modeling of pathway alterations and molecular interactions between multiple biological layers. We trained and evaluated diverse machine learning-based models, differing in the incorporated features and learning algorithms on a cross-omics dataset encompassing mRNA, miRNA, and protein expression profiles obtained from rat liver samples treated with a heterogeneous set of substances. Most of these compounds could be unambiguously classified as genotoxic carcinogens, non-genotoxic carcinogens, or non-hepatocarcinogens based on evidence from published studies. Since mixed characteristics were reported for the compounds Cyproterone acetate, Thioacetamide, and Wy-14643, we reclassified these compounds as either genotoxic or non-genotoxic carcinogens based on their molecular profiles. Evaluating our toxicogenomics models in a repeated external cross-validation procedure, we demonstrated that the prediction accuracy of our models could be increased by joining the biomarker signatures across multiple biological layers and by adding complex features derived from cross-platform integration of the omics data. Furthermore, we found that adding these features resulted in a better separation of the compound classes and a more confident reclassification of the three undefined compounds as non-genotoxic carcinogens.

## Introduction

The current gold standard for evaluation of the carcinogenic potential of newly developed drugs and other chemical compounds is the 2-year chronic rodent bioassay. This assay requires daily administration of the tested compound to rats or mice of both sexes and close survey of the animals for signs of toxicity and neoplastic lesions (see OECD Test Guideline 451). During preclinical safety assessment, substances showing a carcinogenic potential at a late stage may lead to substantial losses for the pharmaceutical and chemical industry. A mechanistic distinction is made between genotoxic carcinogens (GCs), which form DNA adducts and cause direct DNA damage, as opposed to non-genotoxic carcinogens (NGCs), for which a wide variety of alternative hepatocarcinogenic mechanisms have been described [1]–[3]. While GCs can be identified early by means of *in vitro* genotoxicity assays (e.g., Ames test), no short-term assay exists for the detection of NGCs.

Several groups have reported application of toxicogenomics methods for prediction of the outcome of chronic bioassays based on gene expression profiles compiled from short-term *in vivo* studies. Most studies published in this field focused on mRNA expression profiling and employed machine learning algorithms or statistical methods to predict the carcinogenic class of compounds based on characteristic expression patterns, called signatures [4], [5]. The classification outcomes may then be used to prioritize environmental and/or industrial chemicals for further exploration in chronic carcinogenicity bioassays [6]. Furthermore, the toxicogenomics approach can deliver complementary mechanistic insights, as specific molecular profiles can be associated with toxicological phenotypes and adverse effects observed in animal studies [5]. Along these lines, Ellinger-Ziegelbauer *et al.* revealed characteristic changes in the expression of mechanistically related genes, which allowed for discriminating GCs from NGCs in male Wistar-Hanover rats [7]. While a strong DNA damage response was observed upon GC treatment, the NGC- induced genes were indicative of increased cell cycle progression [7]. In a follow-up study, the authors constructed prediction models based on Support Vector Machine (SVM) classifiers trained on a larger set of compounds and thereby demonstrated the potential of toxicogenomics approaches for the early assessment of the carcinogenic risk [8]. More recently, Uehara *et al.* proposed two mRNA signatures for the detection of certain classes of NGCs based on Affymetrix gene expression data from Sprague-Dawley rat liver samples, which are deposited at the large toxicogenomics database TG-GATEs [9]. While the first signature captured transcriptional changes present upon single NGC exposure after 24 hours [10], the second one contains probe sets which are specifically deregulated after 4 weeks of repeated NGC administration [11]. Furthermore, an approach using environmental chemicals found that carcinogens in general can be detected with higher specificity if longer dosing periods are used in animal studies [6], [11]. These findings suggest that the exact compound class to be predicted and the time point(s) at which expression profiles are generated should be well defined and considered together.

In addition to global mRNA profiling data, miRNA, and protein expression data have previously been generated for toxicogenomics applications [12], [13]. While microarrays and RNA-seq are typically used for transcriptional profiling, shotgun proteomics is commonly employed for global protein profiling [14]. A review of the state-of-the-art high-throughput techniques for holistic molecular profiling including references to published applications in the field of toxicogenomics was recently provided by Khan *et al.* [14].

In general the ability to predict a compound's carcinogenic potential based on short-term expression profiles would be a clear step forward concerning reduction of time, animals, and monetary requirements in chemical and drug development. Building on published toxicogenomics studies that mostly focused on mRNA expression and used individual genes as predictive features, the approaches presented here introduce two novel concepts: first, the integration of omics data across platforms that interrogate different biological layers (mRNA, miRNA, and protein expression) and second, the abstraction from individual signature genes to higher-order levels, such as pathway enrichments or molecular interactions. This holistic approach, which integrates multiple global omics approaches, was currently also proposed by Khan *et al.* [14].The classification performance of our novel methodologies was evaluated on a dataset comprising mRNA, miRNA, and protein expression profiles from liver samples of male Wistar rats exposed to GCs, NGCs, or non-hepatocarcinogens (NCs) for up to 14 days. In a cross-validation experiment, we demonstrate that the predictive power of traditional mRNA signatures can be increased by adding complementary omics-based features obtained from profiling other molecular levels. We show that the classification performance can be further improved by additionally providing the prediction models with complex features derived from integrated analyses of multi-level omics data.

## Methods

### Ethics statement

The experimental protocol was reviewed and approved by the Institutional Animal Care and Use Committee (IACUC) of the Institute of Toxicology, Bayer, Stilwell, KS, U.S.A, for compliance with the Federal Animal Welfare Act (1988): 7 U.S.C.2131 et seq. as well as the National Research Council's (NRC) Guide for the Care and Use of Laboratory Animals (National Academy Press, 1996). General clinical observations, including observations for moribundity and mortality, were performed at least daily to monitor the general overall health status of the animals and to minimize suffering. For necropsy, the animals were anesthetized in a CO2 chamber and blood was drawn by cardiac puncture. Finally, animals were exsanguinated by cutting the diaphragm.

### Animal study

Male Wistar Hanover rats (Crl:WI[Gl/BRL/Han]IGS BR) from Charles River Laboratories, Inc. (Raleigh, NC) were maintained on certified rodent chow (Purina Mills Certified Rodent Diet 5200) ad libitum in individual suspended stainless steel wire-mesh cages. The animals were kept under controlled temperature (18 to 26°C), humidity (30 to 70%), and lighting (12 h light – dark cycle) and were acclimated for a minimum of 6 days. 8 to 10 week old animals were assigned to dose groups (5 rats/group) by weight using a weight stratification-based computer program. Substances were administered by gastric gavage for up to 14 days (in a volume of 5 ml/kg body weight/day) based on the group mean weekly body weight for each dose group. Test substances were suspended (W/W) in either corn oil, or a 0.5% (W/V) Carboxymethyl Cellulose (CMC)/DI water preparation (5 g CMC/1 liter DI water). To maintain a homogenous suspension during dosing, a magnetic stir-plate was used if needed. Dimethylnitrosamine (Sigma, St. Louis, MO; CAS 62-75-9; purity >98%; 4 mg/kg/d), C.I. Direct Black (Chlorazol Black, Sigma, St. Louis, MO; CAS 1937-37-7; purity 29% carbon, 146 mg/kg/d), and Cyproterone acetate (Sigma, St. Louis, MO; CAS 427-51-0, purity 97.3%, 100 mg/kg/d) were prepared using corn oil as vehicle. Thioacetamide (Sigma, St. Louis, MO; CAS 62-55-5, purity 99.2%, 19.2 mg/kg/d), Wy-14643 (TCI America, Portland, OR; CAS 50892-23-4; purity 100%; 60 mg/kg/d), Phenobarbital (Sigma, St. Louis, MO; CAS 50-06-6, purity >99%, 80 mg/kg/d), Piperonylbutoxide (Sigma, St. Louis, MO; CAS 51-03-6; purity 88.5%; 1200 mg/kg/d), Dehydroepiandrosterone (Sigma, St. Louis, MO; CAS 53-43-0, purity 100%, 600 mg/kg/d), Acetamide (Sigma, St. Louis, MO; CAS 60-35-5, purity 99%, 3000 mg/kg/d), Methapyrilene hydrochloride (Sigma, St. Louis, MO; CAS 135-23-9; purity >99%; 60 mg/kg/d), Methylcarbamate (Sigma, St. Louis, MO; CAS 598-55-0, purity 99.2%, 400 mg/kg/d), Diethylstilbestrol (Sigma, St. Louis, MO; CAS 56-53-1; purity 99%; 10 mg/kg/d), Ethionine (Sigma, St. Louis, MO; CAS 67-21-0; purity >99%; 200 mg/kg/d), Cefuroxime (Sigma, St. Louis, MO; CAS 55268-75-2; purity not provided%; 250 mg/kg/d), and Nifedipine (Sigma, St. Louis, MO; 21829-25-4; purity 99%; 3 mg/kg/d) were dosed using Carboxymethyl Cellulose as vehicle. Diethylstilbestrol and Piperonylbutoxide were administered for 1, 3, and 7 days. All other compounds were dosed for 1, 3, 7, and 14 days. Time points selected for evaluation in this report are listed in Table 1. The rationale for dose selection was based on those reported to induce liver tumors in the two-year rat bioassay [8]. From each treatment group three animals that showed at least some changes in the liver as observed by histopathological examination were selected for microarray analysis [8]. Time-matched control groups of equal size treated with the corresponding vehicles methylcellulose (MC) or corn oil (CO), served as a reference to determine the changes in gene expression upon treatment.

**Table 1:** Overview of compounds.

Class	Compound	Short name	CAS number	Vehicle	Dosing time [d]	Dose [mg/kg/d]	IARC class
**Genotoxic carcinogens (GC)**	C.I Direct Black	CIDB	1937-37-7	CO	7	146	1
	Dimethylnitrosamine	DMN	62-75-9	CO	7	4	2A
**Undefined compounds**	Cyproterone acetate	CPA	427-51-0	CO	14	100	-
	Thioacetamide	TAA	62-55-5	CMC	7	19.2	2B
	Wy-14643	WY	50892-23-4	CMC	3	60	-
**Non-genotoxic carcinogens (NGC)**	Phenobarbital	PB	50-06-6	CMC	14	n/a	2B
	Piperonylbutoxide	PBO	51-03-6	CMC	3	1200	3
	Dehydroepiandrosterone	DHEA	53-43-0	MC	14	600	-
	Acetamide	AA	60-35-5	MC	14	3000	2B
	Methapyrilene HCl	MPy	135-23-9	MC	7	60	-
	Methylcarbamate	Mcarb	598-55-0	MC	14	400	3
	Diethylstilbestrol	DES	56-53-1	MC	3	10	1
	Ethionine	ETH	67-21-0	MC	14	200	-
**Non-carcinogens (NC)**	Cefuroxime	CFX	55268-75-2	CMC	14	250	-
	Nifedipine	Nif	21829-25-4	CMC	14	3	-

The table lists all compounds that were repeatedly administered to male Wistar rats for a time span of up to 14 days. The time point where the strongest deregulation was observed on the mRNA level was determined for each compound individually based on results from a preceding study by Ellinger-Ziegelbauer *et al.* [8]. For each compound, the corresponding short name, which was used in the figures and text, is denoted. Furthermore, the vehicle and dose used for oral application is listed.

### Messenger-RNA expression profiling

To monitor global changes in mRNA expression, biotin-labeled cRNA samples were prepared with a starting amount of 5 µg of total RNA according to the manufacturer's instructions (Affymetrix, USA; GeneChip Expression Analysis 701194 Rev.1) and hybridized on Affymetrix GeneChip RAE230A arrays. Fluorescent images of the GeneChips were captured with the Affymetrix GeneChip Scanner 3000. Raw data image files (DAT) were converted into CEL files using Affymetrix Microarray Suite (MAS) 5.0 in which the scan data from the 36 pixels per oligo set are summarized as one intensity value. The RAE230A array used in this study contains 15.866 probe sets, corresponding to approx. 5399 annotated rat genes and 10467 expressed-sequence tags (EST).

For a complete description of the experimental protocol the reader is referred to a former publication from Ellinger-Ziegelbauer *et al.* [8]. The quality of the raw data was assessed based on diverse plots and statistics implemented in the package *arrayQualityMetrics* for R/Bioconductor [15], [16]. No experimental problems were detected and all chips were found to have sufficient quality. Background correction, normalization between arrays, and probe summarization were performed based on the Robust Multi-chip Average (RMA) technique implemented in the *affy* package for R/Bioconductor.

### Micro-RNA expression profiling

100 ng total RNA was end-labeled using the Agilent miRNA labeling kit (Agilent p/n 5190–0456). End-labeled miRNA samples were purified using Qiagen PCR clean up columns. Labeled miRNA samples were hybridized according to the Agilent miRNA Microarray System with miRNA Complete Labeling and Hyb Kit Protocol G4170-90011 V2.2 October 2009 using reagents contained in the Agilent miRNA labeling kit. Prior to array hybridization, hybridization mixtures were denatured at 100°C for 5 minutes. Hybridization was carried out at 20 RPM at a temperature of 55°C for 20 hours before washing in Agilent Gene Expression Wash Buffer 1 and Agilent Gene Expression Wash Buffer 2 (Agilent p/n 5188–5327). Hybridization, scanning, and image analysis were performed using the Agilent DNA Microarray Scanner equipped with extended dynamic range (XDR) software according to the Agilent miRNA Microarray System with miRNA Complete Labeling and Hyb Kit Protocol G4170–90011 V2.2 October 2009. Agilent Feature Extraction Software v10.7 was used for data extraction from raw microarray image files.

As previously done for the mRNA expression data, we ensured sufficient quality of the raw data by performing quality checks implemented in the R library *arrayQualityMetrics*, which supports a wide variety of microarray platforms [15]. In order to compensate for experimental artifacts and variation between arrays, the raw data was preprocessed using a variant of the RMA algorithm that was specifically implemented for Agilent miRNA microarrays by Lopez-Romero *et al.* [17]. The RMA implementation from the *AgiMicroRna* library for R/Bioconductor was used without the initial background correction step, as recommended by Lopez-Romero *et al.*, who recently demonstrated that this normalization technique facilitates a more accurate estimation of miRNA expression levels than the method suggested by the array manufacturer [18]. Oligos that were not expressed in any of the profiled samples were not considered in further analysis steps. In order to link miRNAs to global gene expression, putative regulatory interactions were inferred between miRNAs and experimentally confirmed and predicted target mRNAs. For this purpose, a non-redundant set of validated miRNA/mRNA interactions was compiled by combining information from the databases TarBase v5.0c [19], miRTarBase v2.4 [20], and miRecords v3 [21]. Predicted miRNA targets were collected with the miRNA target prediction tools ElMMo v5 [22], DIANA-microT v4.0 [23], and TargetScan v5.2 [24].

### Protein expression profiling

Protein expression profiling was performed using reverse-phase protein microarrays (RPPAs) on the ZeptoMARK assay platform (Bayer Technology Services, Leverkusen, Germany). Frozen liver tissue (50–80 mg tissue) was weighted into a cryovial and grinded to a fine powder under liquid nitrogen. 8 volumes of lysis buffer were added to the pulverized tissue and lysis was carried out in a rotating mixer for 30 min. Protein concentration of the lysate was determined by a Bradford assay and protein concentration of the lysates was adjusted to 0.3 mg/ml protein. The prepared lysates were used to print RPPAs as described in detail by Pirnia et al. [25]; samples and reference material (BSA labeled with Alexa-647) were spotted (300 pl/spot) on Zeptosens hydrophobic protein microarray chips (Bayer Technology Services, Leverkusen, Germany). Detection of proteins and protein modifications was performed using a direct two-step immunoassay using specific primary antibodies (see supplement). Fluorescence signal was generated using Alexa647-labeled anti-species secondary antibodies (Invitrogen, Darmstadt, Germany), images of the microarrays were taken using the ZeptoREADER microarray imager (Bayer Technology Services, Leverkusen, Germany), and image analysis was performed using the ZeptoVIEW Pro 3.0 software package. Signal intensity for each spot was determined as background-corrected mean intensity with the local background subtracted from the spot intensity and the determined values were normalized over the whole RPA. The weighted mean of replicate sample spots was used for statistical analysis; standard deviation was calculated according to standard error propagation rules from the standard deviations of raw and blank signals.

For the sake of improved interpretability, the blank-corrected normalized signal levels were divided by the median of the corresponding control group samples incubated with the same specific antibody and subsequently log_2_-transformed. If the background signal level, which was determined by exclusive use of the secondary antibody, exceeded the combined signal level of both the primary specific and secondary antibodies when used in combination, the corresponding measurement was considered as a missing value. Missing values, which accounted for approx. 1% of the data, were estimated using k-Nearest-Neighbor imputation.

### Inference of predictive molecular signatures

Predictive signatures were inferred based on the fold changes obtained from multiple omics platforms using recursive feature selection with SVMs (SVM-RFE). SVM-RFE repeatedly trains an SVM on the provided features and assigns a weight for each feature depending on the relevance for the classification [26]. After each training cycle, the least informative features are removed and the training is repeated with the reduced set of features. This process is repeated until the desired signature size is reached.

To find the optimal signature size, we evaluated the predictive power of signatures of different size for each supervised machine learning method used for classification. Signatures containing 5, 10, 15, 20, and 25 features were extracted and evaluated for their predictive power. Based on the accuracies estimated for each of the differently sized signatures, we used spline interpolation to numerically approximate the optimal signature size.

As multiple signatures were extracted for each cross-validation fold and repetition, the individual signatures were merged into a consensus signature. For this purpose, the features were ranked by their average rank in all cross-validation folds and repetitions. The consensus signature was then constructed by selecting the best features until the approximated optimal signature size was reached.

### Calculation of molecular interaction features

**Figure 1:**
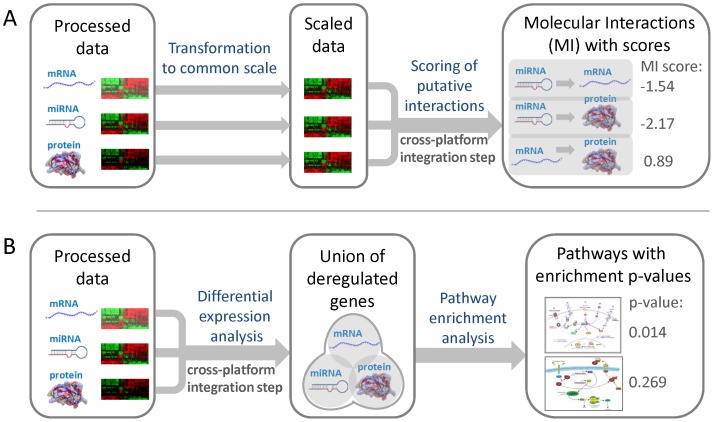
Feature representations used for cross-toxicogenomics prediction models. (A) Molecular interaction features. The processed data from the different platforms, given in the form of log_2_-transformed fold changes, were mapped to the same interval (here: [−1, 1]) using a linear function in order to account for the different dynamic ranges of the platforms. Next, putative interactions between molecules represented on different platforms were inferred based on negatively or positively correlated expression profiles. For miRNAs, all possible interactions to experimentally validated and predicted mRNA targets were considered. Associations between mRNAs and proteins were made based on common gene loci. The connections between miRNAs and proteins can be transitively inferred from the corresponding mRNA interactions. In order to obtain a numeric feature representation, a score was computed for each interaction, which equals the product of the scaled log-ratios calculated for the two interacting molecules. **(B) Pathway enrichment features.** First, differentially expressed features were detected for each platform separately based on appropriate fold change and/or p-value cutoffs. All transcripts and proteins were mapped to the corresponding genes in order to facilitate their association with metabolic and signaling pathways. As miRNAs are typically not contained in canonical pathways, deregulated miRNAs were represented by the genes corresponding to their experimentally confirmed target mRNAs in order to model their impact on pathways. The union of deregulated genes was computed across platforms. Then a hypergeometric test was applied to determine enriched pathways represented by these genes. Finally, a feature vector was constructed, representing the log_10_-transformed p-values obtained for each pathway from the overrepresentation test.

As the regulation of gene expression is orchestrated by the interplay of multiple biological layers, an approach that assesses the relevance of each genomic and proteomic feature individually may be insufficient. In order to account for the highly interconnected nature of gene regulatory mechanisms, we conceived a novel feature representation for multi-level omics datasets, which captures characteristic molecular interactions present upon treatment with a specific class of compounds (Figure 1A).

As the dynamic range of differential expression varies between platforms interrogating different biological layers, we propose to transform the log-ratios, i.e., log_2_(fold changes), to a common scale. Furthermore, the scaling of the data also ensures that each platform contributes equally to the score computed for a certain molecular interaction. All log-ratios *x* were mapped to the same interval using the linear function 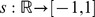 with 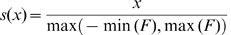, where 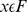 and 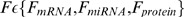 is the set of all log-ratios observed for a certain platform. Next, we computed a score for each putative interaction between two molecules (e.g., miRNA and target mRNA), which equals the product of the scaled log-ratios: 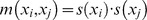, where 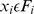 and 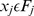 with 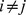. This molecular interaction (MI) score is expected to be close to 1 if correlated expression is observed, e.g., for an mRNA and the corresponding protein product. If the MI score is close to −1 this indicates a strong anticorrelation, which may be observed between a miRNA and one of its target mRNAs. We considered negative correlations between miRNAs and their targeted mRNAs, positive correlations between mRNAs and translated proteins, and negative correlations between miRNAs and proteins that are translated from the targeted mRNAs. Both validated mRNA targets from curated databases (TarBase v5.0c [19], miRTarBase v2.4 [20], miRecords v3 [21]) and predicted mRNA targets inferred by prediction tools (ElMMo v5 [22], DIANA-microT v4.0 [23], TargetScan v5.2 [24]) were used for feature construction.

### Calculation of pathway enrichment features

The basic idea of this feature type is to generate a more robust representation of the molecular signature of a compound by abstraction from genes to pathways (Figure 1B). To this end, we propose to first detect the differentially expressed transcripts/proteins by defining appropriate filter criteria. In this study, we considered genes with an absolute fold change above 2.5 as significantly differentially expressed. Next, we combined the lists of deregulated genes by computing the union across platforms. For this purpose, all profiled mRNAs and proteins were mapped to their corresponding genes, which can in turn be attributed to pathways. Since miRNAs are *a priori* not contained in canonical pathways (e.g., from KEGG, Ingenuity, etc.), their regulatory influence on pathways was modeled based on experimentally validated interactions to known target mRNAs corresponding to pathway nodes. Overrepresented pathways for this combined gene list, derived from deregulated mRNAs, miRNA targets, and proteins, were detected with a hypergeometric test. Given a universe where the union of all pathways contains *N* genes of which *M* are in the pathway of interest, and the combined list containing *n* genes of which *m* are contained in the pathway, the p-values were computed according to the following formula:
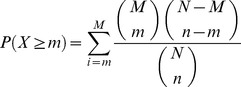



The p-values were then transformed into enrichment scores, which correspond to the -log_10_(p-value). Finally, a vector composed of the enrichment scores of each pathway was employed as a numeric feature representation of pathway alterations induced by compound treatment. The higher the enrichment score computed for a certain pathway, the more significant is the overrepresentation of genes from the combined list in this pathway. The feature vectors were constructed based on different sets of canonical pathways extracted from the databases KEGG [27], Reactome [28], and BioCarta [29]. Associations from genes to pathways were derived with the corresponding metadata packages available for R/Bioconductor [16].

### Validation of prediction models

We ensured unbiased parameter tuning and evaluation on independent test compounds by employing a 2×2-fold nested, stratified cross-validation procedure. In order to obtain a more robust estimate of the classification performance, 10 repetitions were performed with different random splits of the data. The prediction accuracy that can be achieved with different feature representations derived from the expression profiles of the compounds was assessed based on the average area under the ROC curve observed for a representative selection of machine learning methods that are prevalent in toxicogenomics applications. We employed linear Support Vector Machines (SVM), Random Forests (RF), Neural Networks (NN), Bayesian Generalized Linear Models (BGLM), and Principal Component Regression (PCR). We included PCR because a good separation of classes was observed in a principal component analysis (PCA) of the data, and because the method achieved comparable accuracy than the other methods on the here evaluated classification problems. We have also analyzed the optimal number of principal components used for classification with PCR (Figure S1). RF, NN, BGLM, and PCR are implemented in the *caret* library available for R [30]. The SVM was used via the R interface provided by the SHOGUN machine learning toolbox [31].

## Results

### Classification performance of omics signatures

**Figure 2:**
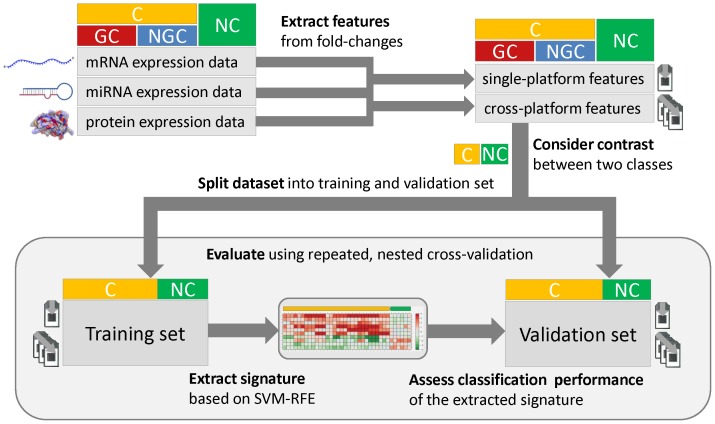
Workflow used for signature extraction and evaluation of classification performance. For the multi-level omics data available in this study, which include mRNA, miRNA, and protein expression profiles of diverse compounds, fold changes were calculated for each gene and sample that could be confidently assigned to a certain compound class (C: carcinogens, GC: genotoxic carcinogens, NGC: non-genotoxic carcinogens, NC: non-carcinogens). While traditionally used single-platform features simply correspond to fold changes observed on each specific biological level, cross-platform features capture molecular interactions and pathway alterations, which can be inferred by integrating omics data across multiple levels. For each class contrast (e.g., C vs. NC) of interest, the dataset was split into a training set and a validation set. Using the SVM-RFE feature selection technique, a predictive signature for class discrimination was extracted, which was then used to predict the carcinogenic class of the samples in the validation set. By embedding this process into a 2-fold cross-validation with 10 repetitions that use different random splits of the data, the classification performance can be robustly estimated based on the mean area under the ROC curve.

With the goal to identify molecular signatures that may allow early prediction of a carcinogenic risk associated with a compound, we inferred mRNAs, miRNAs, and protein expression signatures correlated with the toxicological classes of the compounds which induced these signatures in the liver of rats treated for up to 14 days. In addition, a combined signature was obtained by merging the signatures extracted from each individual platform. Furthermore, we compiled signatures based on putative molecular interactions (MI features) observed between two molecular layers, as well as based on pathways enriched with genes/proteins that were detected as deregulated (PE features). These two types of cross-platform features (Figure 1) were joined in different arrangements with the combined single-platform signatures in order to create different types of hybrid signatures. Signature inference and evaluation were performed in a 2×2 cross-validation setting for three different class contrasts (C:NGC+GC vs. NC, NGC vs. GC, NGC vs. NC) using five established supervised classification methods (Figure 2). The evaluation process was repeated ten times with different random cross-validation splits and then ROC curves were generated based on the prediction scores obtained in each run (Figure S2). In order to assess the prediction accuracy, we calculated the average area under the curve (AUCs) for each contrast, classifier, and signature type.

**Figure 3:**
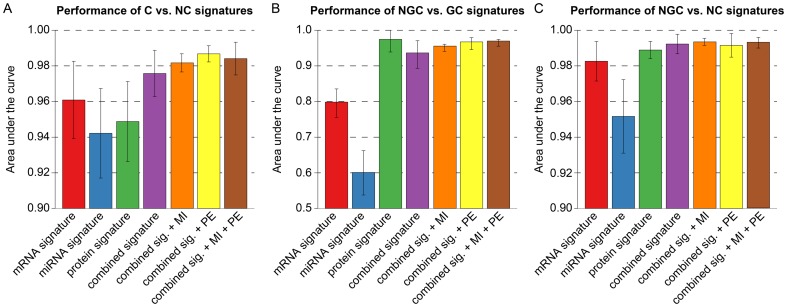
Classification performance for different class contrasts depending on signature types. The bar plots correspond to the average area under the ROC curve obtained from five widely used supervised classification methods (SVM, RF, NN, PCR, and BGLM). Before averaging across classifiers, the prediction scores were integrated across repetitions and cross-validation folds. Each column corresponds to a certain signature type, which may be composed of different modules. The combined signature contains all predictive features from the mRNA, miRNA, and protein signatures. +MI and +PE indicate the additional use of molecular interaction and pathway enrichment features, respectively. Bar plots were generated for (**A**) C vs. NC classification, (**B**) NGC vs. GC classification, and (**C**) NGC vs. NC classification.

Since in most cases an increased average AUC was observed for the combined signatures, we can conclude that composite signatures derived from multiple platforms, may allow for a more accurate compound classification than the traditionally used single-platform signatures (Figure 3). The only exception was the protein signature for the NGC vs. GC contrast, which outperformed the corresponding combined signature for this special setting (Figure 3B). In comparison to the mRNA signature (the current standard approach in toxicogenomics) and the miRNA signature, the combined signature provided a consistent improvement of AUCs. Interestingly, the integration of our novel MI and PE features led to a further increase in terms of the average AUC. For C vs. NC and NGC vs. GC discrimination, the use of hybrid signatures including the new features consistently resulted in higher classification accuracy than using all single-platform signatures even if combined (Figure 3A, C). For the NGC vs. NC contrast the effect was very small, but still the signatures containing the MI features achieved a slightly better AUC than the combined signature. The combination of all signatures (i.e., the combined signature plus the MI and PE features) achieved the best AUCs in two of the three contrasts and the second best in the C vs. NC contrast. Furthermore, the prediction accuracy of the single-platform signatures was found to heavily depend on the contrast under evaluation. In the NGC vs. GC contrast, both the mRNA and the miRNA signatures performed significantly worse than in the other two contrasts, whereas the protein signature was inferior in terms of accuracy in the C vs. NC contrast. On the contrary, we observed that the combined signature and the hybrid signatures complemented by the MI and PE features achieved high (AUC>0.95) and robust AUCs independent of the evaluated class contrast.

### Predictive features for toxicogenomics models

**Figure 4:**
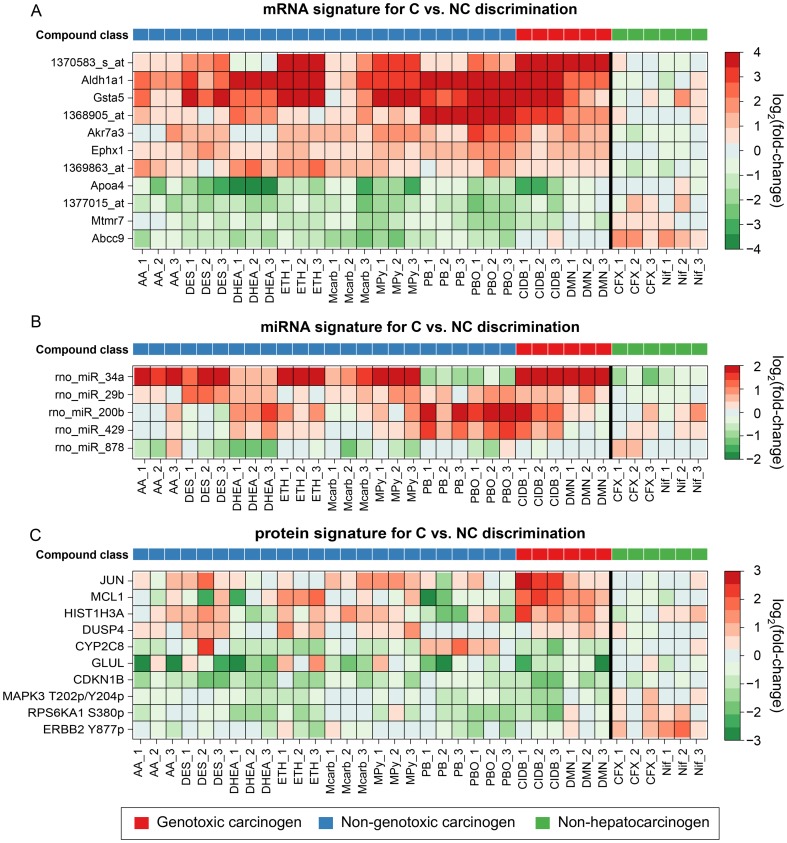
Heatmap plots of single-platform signatures for C vs. NC classification. The heatmaps depict characteristic expression patterns observed in livers of rats after exposure to diverse rodent liver carcinogens and non-carcinogens. A selection of signature molecules is shown for each profiled molecular level: (**A**) mRNA expression, (**B**) miRNA expression, and (**C**) protein expression. In each heatmap, rows correspond to signature molecules and columns correspond to liver samples from differentially treated rats. The bold vertical lines separate the carcinogens from the Non-carcinogens. Plotted are the log_2_(fold changes), where red indicates up-regulation and green indicates down-regulation (see color keys). The color bar on top refers to the compound class (see legend).

For each classifier, consensus signatures were generated by combining the feature rankings inferred from random data splits, which correspond to different repetitions of the cross-validation procedure. The extracted signatures are shown in Table S1 for C vs NC discrimination, Table S2 for NGC vs GC discrimination and Table S3 for NGC vs NC discrimination. The most informative genes and proteins for distinguishing C from NC are illustrated in the heatmaps in Figure 4. A correlation between gene expression and carcinogenicity could be observed for the mRNA signature (Spearman's ρ>0.5). However, both the informative miRNAs and proteins do not show clear, carcinogen-specific expression patterns. In particular, some of the top miRNA and protein features (e.g., rno_miR_34a and CYP2C8) show uncharacteristic expression changes in the PB and PBO samples (see Figure 4B,C). These findings are consistent with the fact that a lower classification performance was observed for these two signature types (Figure 3). The heatmaps showing the top features of the single platform signatures for the NGC vs. GC and NGC vs. NC contrast are shown in Figure S3 and S4, respectively.

The most informative mRNA markers for C vs. NC discrimination include various genes known to be involved in cellular responses to carcinogenic exposure, e.g., *Gsta5*, *Aldh1a1* [32], [33], *Ephx1* [34], and *Akr7a3* [35], which are detoxifying enzymes, for instance, in the context of oxidative stress. Some miRNAs identified in our experiments as early markers for prediction of a carcinogenic potential are also mentioned in connection with cancer in the literature, e.g., rno-miR-34a and rno-miR-200b [36], [37]. Furthermore, the protein signature for C vs. NC discrimination also contains proteins that have been linked to carcinogenesis, e.g., JUN [38], GLUL [39], and CDKN1B [40].

**Figure 5:**
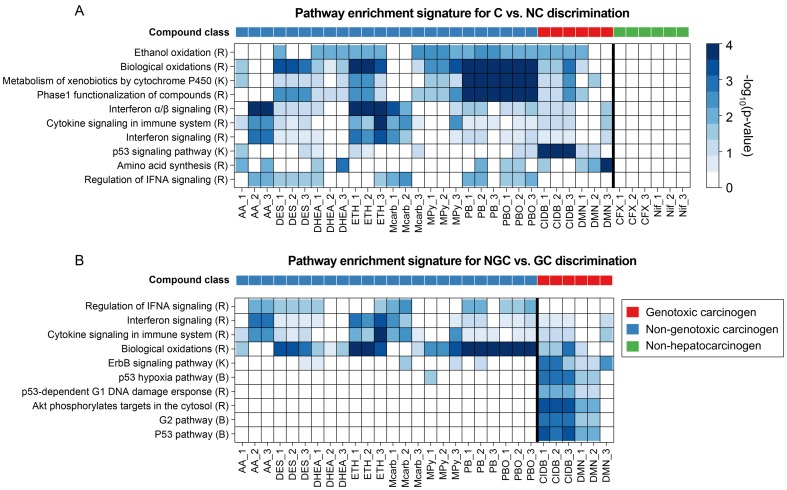
Heatmap plots of pathway enrichment signatures. The heatmaps depict overrepresentation of genes involved in relevant pathways among the genes deregulated in liver upon treatment of rats with a certain compound. Pathways relevant for compound classification were selected by SVM-RFE for different class contrasts: (**A**) C vs. NC and (**B**) NGC vs. GC. The rows correspond to canonical pathways from the databases Reactome (R), KEGG (K), or BioCarta (B) and the columns correspond to samples. The bold vertical lines separate sample classes. The color of each cell refers to the -log_10_(p-value) obtained from a hypergeometric overrepresentation test and indicates the significance of a certain pathway enrichment (see color key). The color bar on top of each heatmap denotes the carcinogenic class (see legend).

As has been done for the single-platform signatures, consensus signatures were generated based on the MI and PE signatures extracted for each run. The most informative pathways for the C vs. NC and NGC vs. GC contrasts are depicted in the heatmap in Figure 5.

The top PE features for the discrimination of carcinogens from non-carcinogens were highly specific for carcinogens and showed no enrichment at all in the non-carcinogens (Figure 5A). For the discrimination of genotoxic and non-genotoxic carcinogens, specific pathways could be inferred for both genotoxic and non-genotoxic carcinogens (Figure 5B). Only one of the three DMN samples shows an uncharacteristic enrichment pattern. Except for this outlier, the pathways altered in GC-exposed rats are highly specific, showing strong enrichment in the genes deregulated upon GC, but not upon NGC treatment. Consistent with our expectations, various pathways related to *p53*, which is a key gene in the cellular DNA damage response, were selected as GC-specific pathway features [41]. The NGC-specific pathways include cytokine and interferon signaling pathways, which have been associated with non-genotoxic carcinogenesis before [42]. These pathways are also specific for the discrimination of NGCs and NCs (Figure S5).

**Figure 6:**
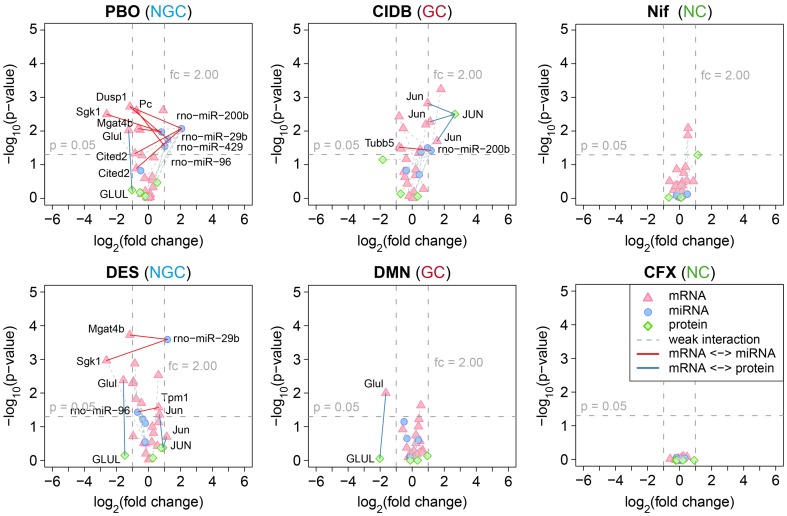
Volcano plots of molecular interaction signatures. Shown are volcano plots for two representative compound profiles of each of the three compound classes (i.e., NGC, GC, and NC). The plots represent putative molecular interactions between different molecular layers, which were found to be predictive for C vs. NC classification. For each interacting molecule (i.e., mRNA, miRNA, or protein) the strength of its differential expression was assessed in terms of the log_2_(fold change) and plotted against its significance, which is given by the FDR-corrected log_10_(p-value) obtained from a moderated t-test. Different shapes and colors denote different types of molecules (see legend). Colored edges were used to highlight molecular interactions for which a positive or negative correlation was observed between two molecule types. We considered correlations in the expression profiles of miRNAs and their experimentally confirmed or predicted mRNA targets, as well as between mRNAs and proteins sharing the same genomic locus. As a formal criterion for a putative molecular interaction, we required that for both interaction partners a 50% increase or decrease in expression could be observed relative to the controls.

The MI consensus signature for the discrimination of carcinogenic and non-carcinogenic compounds is depicted in Figure 6. We highlighted interactions where both interacting molecules were at least 1.5-fold (50%) up- or downregulated. While a negative correlation in the expression pattern was expected for putative miRNA-mRNA interactions (i.e., upregulated miRNA and downregulated mRNA and vice-versa), a positive correlation was expected for mRNAs and proteins corresponding to the same gene. Using SVM-based recursive feature elimination (SVM-RFE), we identified diverse putative interactions specific for carcinogens. These interactions also involved several genes that have previously been associated with carcinogenesis in the rat, such as *Glul* [39], *Dusp1* [43], *Jun* [38], *Sgk1* [44], and *Mgat4b* [45]. Correlated changes at the transcription and translation level that were exclusively observed after treatment with rat hepatocarcinogens were found for the genes *Glul* and *Jun* (Figure 6 and S6). Putative interactions that were specifically found for NGCs include the anti-correlation between the miRNA rno-miR-29b and its potential target mRNAs, *Sgk1*, and *Mgat4*. For the non-carcinogens, we did not observe any putative interactions affecting multiple molecular layers.

In summary, the extracted cross-platform signatures showed that specific pathways and informative molecular interactions could be identified which were characteristically affected after short-term treatment of rats with hepatocarcinogens in general, as well as with non-genotoxic and genotoxic carcinogenesis. Furthermore, we demonstrated that using these complex markers as supplementary features for the prediction of the compound class resulted in improved cross-validation performance when compared to traditional single-platform-based signatures.

### Toxicogenomics-based classification of undefined compounds

The three compounds CPA, TAA, and WY are generally described as non-genotoxic rodent hepatocarcinogens, yet due to inconclusive results from genotoxicity assays, one could also consider a genotoxic carcinogenic mode of action concerning rodent hepatocarcinogenesis. For instance, although a negative Ames test indicating a non-genotoxic mode of action with respect to carcinogenicity was reported for CPA [46], Martelli *et al.* reported a positive micronucleus test in female rats [47]. For TAA, the Ames test was also negative, while mixed results were observed in a micronucleus test for genotoxicity [48], [49]. WY is classified as an NGC belonging to the subclass of peroxisome proliferators with respect to rodent hepatocarcinogens [50]. However, Deutsch *et al.* reported a positive result in a Comet assay for WY [51], and clastogenicity was observed in two cell types, albeit at near cytotoxic doses [52]. Therefore, these compounds were not considered as sufficiently and reliably labeled substances suited for training and validation of our prediction models. Instead, we used the signatures inferred from the compounds that could be clearly assigned to a carcinogenic mode of action class to classify these three compounds, employing the same set of classifiers used to assess the prediction accuracy of the inferred signatures. In addition, a principal components analysis (PCA) was performed by transforming the vector of signature features for each compound into a two-dimensional space spanned by the two principal components explaining most of the variance in the data. This should facilitate comparison of the complex, high-dimensional expression patterns observed for the different compounds.

**Figure 7:**
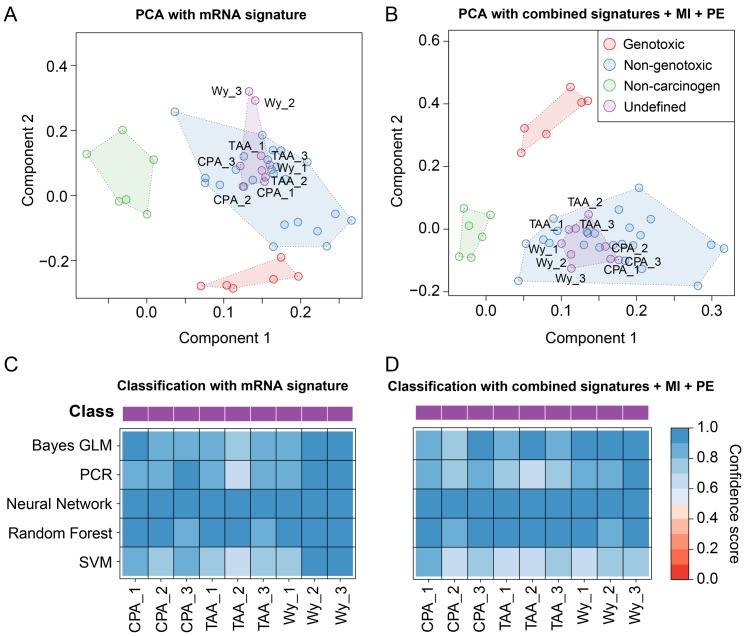
Classification of undefined compounds. (**A**) Samples are represented based on the protein signature for NGC vs. GC discrimination. The corresponding fold changes were PCA-transformed and plotted in a lower-dimensional space spanned by the first two principal components. The color of the spheres corresponds to the class of the administered compounds with respect to their hepatocarcinogenic properties in rats. Clusters of rat liver samples after treatment of rats with compounds of the same class are highlighted by means of transparent polygons. (**B**) Similar plot as in (A), but instead of using the protein signature for sample representation, all single-platform (mRNA, miRNA, protein) and cross-platform signatures (PE, MI) were combined. (**C**) The heatmap displays the confidence scores obtained from five different machine learning methods that were used to classify the undefined compounds CPA, TAA, and WY as either NGC or GC. The confidence scores are [0, 1]-scaled and correspond to the probability that a certain sample was treated with an NGC (see color key). (**D**) Similar illustration as in (C) obtained from an SVM classifier trained on all signatures combined.

The PCA plots resulting from two different signatures for NGC vs. GC discrimination are shown in Figure 7A–B. The PCA plot in Figure 7A is generated from the mRNA expression signature, since mRNA signatures have generally been used before for such classification problems. Although the three classes – GC, NGC, and NC are separated based on mRNA expression only, the GC and NGC class are relatively close together, and the WY samples are placed outside of these three classes. On the contrary, the PCA based on the signature incorporating all single-platform (mRNA, miRNA, and protein) and cross-platform features (PE and MI) showed a clear separation of the compound classes (see Figure 7B). Furthermore, all undefined compounds were enclosed in the NGC cluster, and thus could be clearly classified with our toxicogenomics-derived signature. From Figure 7C–D and Figure S8 it becomes obvious that the PCA-based classification of WY, CPA, and TAA as NGCs is in accordance with the classification achieved with the supervised learning algorithms. The classification solely based on the protein and miRNA signature, respectively, is inconsistent for some of the CPA and TAA samples and on average only possible at a lower confidence level (Figure S8). However, after training of the prediction models on all combined features, high confidence scores and a robust classification across all employed learning algorithms were achieved. In conclusion, all undefined compounds were classified as non-genotoxic carcinogens based on their molecular profiles. These results are also consistent with the outcomes of diverse carcinogenicity assays which were performed for WY, TAA, and CPA [46], [48], [50].

## Discussion

In this study, we propose a new approach for prediction of potential chronic toxicity of chemicals based on expression data obtained from multiple omics platforms after short-term treatment. Prediction of the compound class after short-term treatment may facilitate hazard assessment during preclinical testing and may allow prioritization of chemicals with respect to their cancerogenic potential in chronic bioassays. The current standard approach in toxicogenomics is to use microarrays to screen for differential expression of mRNAs and infer signatures based on fold changes between treated and non-treated animals. Here, we argue that the inclusion of other omics data, e.g., miRNA expression or protein abundance, may increase the predictive power of the inferred signatures. Further, we introduce two new feature representations developed specifically for multi-platform omics data, the molecular interaction (MI) features and the pathway enrichment (PE) features.

With extensive cross-validation, we evaluated the predictive power of single-platform signatures as well as hybrid signatures complemented with cross-platform features on a dataset containing mRNA, miRNA, and protein expression data for 8 NGCs, 2 GCs, and 2 NCs concerning rodent hepatocarcinogenicity. In addition, all signatures were used to classify three rodent hepatocarcinogens with insufficiently defined mode of actions. The dataset used for the evaluation is publically available from the NCBI Gene Expression Omnibus (GEO) repository under the accession number GSE53085 [53], [54].

In comparison to the signatures inferred solely from the mRNA data, we observed an increase in predictive power when combining signatures across multiple platforms for all evaluated class contrasts (C vs. NC, NGC vs. GC, and NGC vs. NC). In addition, we found that the inclusion of complex cross-platform features capturing molecular interactions or pathway alterations led to a further improvement of the classification performance. In general, the combined single-platform signatures with MI and PE features were superior to the single-platform signatures in terms of prediction accuracy, except for the NGC vs. GC contrast, where the protein signature performed slightly better. However, visual inspection of the informative features by PCA revealed a much better separation of classes for the cross-platform signatures (Figure S7). Furthermore, a potential bias may arise from the fact that not the complete proteome, but only a selection of 158 proteins proposed by domain experts were profiled in our experiments. Thus, we hold the opinion that in following studies the predictive power of protein signatures should be assessed on a richer set of compounds based on global protein expression data. Altogether, while a high variability was observed for the classification performance achieved with individual single-platform signatures, a consistently high prediction confidence (AUCs>0.9) was found for models provided with features from multiple molecular layers. Considering all evaluated class contrasts, the combined signature with pathway enrichment and molecular interaction features on average achieved the best classification with the highest prediction accuracies (AUCs>0.95).

In related toxicogenomics studies the accuracy of classifiers trained solely on omics-based features has been compared to models exclusively or additionally provided with features derived from a Quantitative Structure-Activity Relationship (QSAR) approach [55], [56]. Liu *et al.* and Low *et al.* consistently reported that the integration of QSAR-based features did not contribute to an increased classification performance [55], [56]. Since there is a strong heterogeneity concerning the structures and mechanisms of hepatocarcinogenic compounds, the inference of relations between chemical structures and the complex phenotype of cancer is a challenging problem. In this study, we did not pursue this approach since a richer set of compounds would have been required for the construction of generalizable models [57]. Furthermore, a QSAR approach may not be ideally suited for predicting chronic toxicity, as it only considers structural properties of the compounds and does not account for important experimental factors, such as dose level and duration of treatment.

As an alternative to our complex supervised approach for compound class discrimination, we applied clustering to determine if the expression profile based grouping of the compounds agrees with their annotated carcinogenic class (Figure S9). For this purpose, we selected the top 100 mRNAs, miRNAs, and proteins based on their average differential expression, measured in terms of the absolute fold-change averaged across samples. Complete linkage hierarchical clustering with Euclidean distance was applied and the optimal number of clusters between 2 and 8 was determined based on the silhouette score. While animals treated with the same compound mostly clustered together, the cluster result did not reflect the initial grouping of the compounds according to their carcinogenic class (i.e., GC, NGC, or NC). This finding indicated that due to the high mechanistic diversity of the compounds, whereas a classification is possible by using supervised methods.

The single- and cross-platform signatures inferred with our supervised approach have been used to reclassify the compounds with undefined class, i.e., CPA, TAA, and WY. All three compounds are known to be carcinogenic, but some of the genotoxicity test results were inconclusive [46]–[51]. Here, all three compounds were classified as non-genotoxic carcinogens by the majority of classifiers trained on the inferred molecular signatures. Much higher prediction confidence was observed when using the cross-platform signatures, whereas single-platform-based classification remained ambiguous. Again, these findings were consistent with a PCA showing a considerably clearer separation of the classes for the cross-platform signatures (Figures 7 and S7).

In contrast to the standard toxicogenomics approach of inferring signatures from mRNA expression, the combination of single-platform signatures from multiple omics platforms led to improved prediction accuracy. The inclusion of the MI and PE features into these combined signatures offered a true cross-platform integration of the multi-level omics data. These integrated features may yield additional information for supervised learning methods and provide new insights for mechanistic analyses of cross-platform omics data. In addition, the PE features introduce an abstraction from the level of individual genes to higher-order levels, such as complex signaling or metabolic networks. Possibly, this abstraction led to a more robust representation of the complex mechanisms underlying early events during hepatocarcinogenesis in rat. Other relevant mechanistic aspects, which cannot be detected by analyzing each platform individually, may be captured by the here introduced MI features. In consideration of the fact that uncertainty was introduced in the signature extraction process by permitting predicted miRNA-mRNA interactions as potential features, the implication of individual, predictive molecular interactions during hepatocarcinogenesis has to be critically assessed. However, since the focus of this study was the proof of principle that considering multiple molecular layers and interactions between them may increase the reliability of toxicogenomics-based prediction models, the experimental validation and confirmation of the biological relevance of the selected features is beyond the scope of this article.

Due to the broad availability of mRNA expression data for toxicogenomics applications (e.g., from the databases TG-GATEs [9] and DrugMatrix [58]) and the proof-of-principle provided for mRNA signatures, several lists of informative mRNA probe sets have been published for both rat and mouse models concerning prediction of a hepatocarcinogenic potential in short-term studies. Most of these studies focused on the discrimination of non-genotoxic carcinogens from non-carcinogens (NGC vs. NC contrast) or on distinguishing carcinogens from non-carcinogens (C vs. NC contrast). We compared our mRNA signature with previously published signatures from Auerbach *et al.* [6] and Nakayama *et al.* [59] (C vs. NC signatures); Fielden *et al.* [60], Nie *et al.* [61], and Uehara *et al.* [10], [11] (NGC vs. NC signatures); and Ellinger-Ziegelbauer *et al.* [8] (NGC vs. GC vs. NC signature). An overview of the informative genes shared between the published and our signatures is given in Table 2. Our NGC vs. NC signature based on mRNA data contained no common genes with the signatures by Fielden *et al*., Nie *et al*., and Uehara *et al*. (2011), and only one common gene with Uehara *et al*. (2008): *Akr7a3*. However, these studies used other microarray platforms, different compounds, and different treatment durations: Nie *et al*. and Uehara *et al*. (2008) used a single dosage 24 h setting, Fielden *et al*. used a 5 day repeated dosage setting, and Uehara *et al*. (2011) used a longer 28-day exposure. For the C vs. NC signature published by Nakayama *et al*., we found five genes that are also present in our C vs. NC mRNA signature (see Figure S10), while only one common gene is shared between our signature and the 90-day signature published by Auerbach *et al*. The signature published previously by Ellinger-Ziegelbauer *et al*. was in part inferred from the same set of samples analyzed in this study. Hence, we found that 6 probe sets (27%) were in common with our 22-probe-set C vs. NC mRNA signature (see Figure S10) and 7 probe sets (28%) with our 25-probe-set NGC vs. GC signature.

**Table 2:** Comparison with published signatures.

Signature	Contrast	Dosing time [d]	Common genes
**Nakayama ** ***et al.*** ** (2006)** [59]	C vs. NC	28	*Aldh1a1, Akr7a3, Ephx1, Apoa4, Abcb1a*
**Auerbach ** ***et al.*** ** (2010) ** [6]	C vs. NC	90	*Abcb1a*
**Ellinger-Ziegelbauer ** ***et al.*** ** (2008) ** [8]	C vs. NC	up to 14	*Aldh1a1, Akr7a3, Abcc3, Myc, Ces2c, Abcb1a*
**Ellinger-Ziegelbauer ** ***et al.*** ** (2008) ** [8]	NGC vs. GC	up to 14	*Aldh1a1, Cdkn1a1* (2 probes), *Phlda3, Myc, Hsp90ab1, Abcc3*
**Nie ** ***et al.*** ** (2006)** [61]	NGC vs. NC	1	-
**Fielden ** ***et al.*** ** (2007)** [60]	NGC vs. NC	5	-
**Uehara ** ***et al.*** ** (2008) ** [10]	NGC vs. NC	1	*Akr7a3*
**Uehara ** ***et al.*** ** (2011) ** [11]	NGC vs. NC	28	-

The table lists the common genes between our inferred signatures and previously published signatures for the discrimination of carcinogenic and non-carcinogenic chemicals. We compared each published signature with our inferred signature for the same contrast and identified the informative genes common between the signatures.

In summary, we demonstrated that the classification performance of toxicogenomics models benefits from integrating heterogeneous omics data across multiple biological levels. Along these lines, we believe that future toxicogenomics studies may benefit from the additional consideration of other levels, such as metabolomics, relevant DNA mutations, and genome-wide promoter methylation. Furthermore, our work may encourage the maintainers of currently available databases specializing in toxicogenomics (e.g., TG-GATEs [9], DrugMatrix, etc.) to amend the existing repertoire of mRNA expression datasets by the addition of complementary omics data derived from the same biological samples.

## Supporting Information

Figure S1 **Number of selected principle components for PCR.** The histograms show the distribution of the optimal number of principal components selected during the parameter optimization for PCR. Parameter optimization is performed unbiased and independent of the estimation of classification performance for all relevant parameters of the individual classification algorithms. In our experiments, we performed 10 repetitions with different cross-validation splits for each repetition. In each repetition, a 2×2 cross-validation was used, leading to a total of 20 sets of optimized parameters. For PCR, the number of principal components is the only relevant parameter. The distribution of the 20 optimal numbers of principal components for each combination of features is shown in the histograms.
(PDF)

Figure S2 **Cross-validation performance of signatures for compound classification.** (**A**) The receiver operating characteristic (ROC) curves illustrate the predictive power of the signatures inferred for discrimination of carcinogens (C) and non-carcinogens (NC). The left plot illustrates the predictive power of the signature inferred using only mRNA features. The right plot illustrates the predictive power of the signature combining the single-platform signatures for mRNA, miRNA, and protein features as well as the cross-platform molecular interaction (MI) and pathway enrichment (PE) features. For each supervised learning method trained on the extracted signature, one ROC curve has been plotted. The dotted line indicates the chance level, which corresponds to an area under the ROC curve (AUC) of 0.5. The bar plot inside the ROC curve plot indicates the AUC achieved by each classifier. Predictive power was assessed using a 10 times repeated, nested 2×2-fold cross-validation. Prediction scores were scaled linearly to [0,1] and subsequently merged across all folds and repetitions to obtain a single ROC curve. (**B**) Predictive power of signatures for discrimination of non-genotoxic carcinogens (NGC) and genotoxic carcinogens (GC) illustrated as in (A). (**C**) Predictive power of signatures for discrimination of NGCs and NCs illustrated as in (A).
(PDF)

Figure S3 **Heatmap plots of single-platform signatures for NGC vs. GC classification.** The heatmaps depict characteristic expression patterns that were observed in livers of rats after exposure to non-genotoxic or genotoxic rodent hepatocarcinogens. A selection of signature molecules is shown for each profiled molecular level: (**A**) mRNA expression, (**B**) miRNA expression, and (**C**) protein expression. In each heatmap, the rows correspond to signature molecules and the columns correspond to differentially liver samples from differentially treated rats. The bold vertical lines separate the NGCs from the GCs. Plotted are the log_2_(fold changes), where red indicates up-regulation and green indicates down-regulation (see color keys). The color bar on top refers to the carcinogenic compound class (see legend).
(PDF)

Figure S4 **Heatmap plots of single-platform signatures for NGC vs. NC classification.** The heatmaps depict characteristic expression patterns, which were observed in livers of rats after exposure to non-genotoxic hepatocarcinogens and non-carcinogens. A selection of signature molecules is shown for each profiled molecular level: (**A**) mRNA expression, (**B**) miRNA expression, and (**C**) protein expression. In each heatmap, the rows correspond to signature molecules and the columns correspond to differentially treated rat liver samples. The bold vertical lines separate the NGCs from the NCs. Plotted are the log_2_(fold changes), where red indicates up-regulation and green indicates down-regulation (see color keys). The color bar on top refers to the carcinogenic compound class (see legend).
(PDF)

Figure S5 **Heatmap plot for pathway enrichment.** The heatmap depicts overrepresentation of genes involved in relevant pathways among the genes deregulated upon treatment with a certain compound. Pathways relevant for compound classification were selected by SVM-RFE for NGC vs. NC discrimination. The rows correspond to canonical pathways from the databases Reactome (R), KEGG (K), or BioCarta (B) and the columns correspond to samples. The color of each cell refers to the -log_10_(p-value) obtained from a hypergeometric overrepresentation test and indicates the significance of a certain pathway enrichment (see color key). The color bar on top of each heatmap denotes the carcinogenic class (see legend).
(PDF)

Figure S6 **Volcano plots of molecular interaction signatures.** Shown are volcano plots for expression profiles of the six non-genotoxic carcinogens not shown in Figure 6. The plots represent putative molecular interactions between different molecular layers, which were found to be predictive for C vs. NC classification. For each interacting molecule (i.e., mRNA, miRNA, or protein), the strength of its differential expression was assessed in terms of the log_2_(fold change) and plotted against its significance which is given by the FDR-corrected log_10_(p-value) obtained from a moderated t-test. Different shapes and colors denote different types of molecules (see legend). Colored edges were used to highlight molecular interactions for which a positive or negative correlation was observed between two platforms. We considered correlations in the expression profiles of miRNAs and their experimentally confirmed or predicted mRNA targets as well as between mRNAs and proteins sharing the same genomic locus. As a formal criterion for a putative molecular interaction, we required that for both interaction partners a 50% increase or decrease in expression was observed relative to the controls.
(PDF)

Figure S7 **Principal component analysis of signatures for classification of undefined compounds.** (**A**) Samples are represented based on the mRNA signature for NGC vs. GC discrimination. The corresponding fold changes were PCA-transformed and plotted in a lower-dimensional space spanned by the first two principal components. The color of the spheres corresponds to the class of the administered compounds with respect to their hepatocarcinogenic properties in rats. Clusters of rat liver samples after treatment of rats with compounds of the same class are highlighted by means of transparent polygons. (**B**) Similar plot as in (A), but based on the miRNA signature for NGC vs. GC discrimination. (**C**) Similar plot as in (A), but instead of using a single-platform signature for sample representation, all single-platform (mRNA, miRNA, protein) signatures were combined. (**D**) Similar plot as in (A), but instead of using a single-platform signature for sample representation, all single-platform (mRNA, miRNA, protein) signatures and the cross-platform pathway enrichment (PE) signature were combined. (**E**) Similar plot as in (A), but instead of using a single-platform signature for sample representation, all single-platform (mRNA, miRNA, protein) signatures and the cross-platform molecular interaction (MI) signature were combined.
(PDF)

Figure S8 **Reclassification of undefined compounds.** (**A)** The heatmap displays the confidence scores obtained from five different machine learning methods that were used to classify the undefined compounds CPA, TAA, and WY as either NGC or GC based on the mRNA signature for NGC vs. GC discrimination. The confidence scores are [0, 1]-scaled and correspond to the probability that a certain sample was derived from rats treated with an NGC (see color key). (**B**) Similar illustration as in (A) obtained from an SVM classifier trained on the miRNA signature. (**C**) Similar illustration as in (A) obtained from an SVM classifier trained on all single-platform (mRNA, miRNA, protein) signatures combined. (**D**) Similar illustration as in (A) obtained from an SVM classifier trained on all single-platform (mRNA, miRNA, protein) signatures and the cross-platform pathway-enrichment (PE) signature combined. (**E**) Similar illustration as in (A) obtained from an SVM classifier trained on all single-platform (mRNA, miRNA, protein) signatures and the cross-platform molecular interaction (MI) signature combined.
(PDF)

Figure S9 **Cluster analysis of mRNA, miRNA, and protein expression data.** The heatmaps depict the results of a cluster analysis limited to the top 100 differentially expressed (**A**) mRNAs, (**B**) miRNAs, and (**C**) proteins in livers of rats treated with diverse carcinogenic or non-carcinogenic compounds. In each heatmap, the rows correspond to the animals and the columns correspond to the profiled molecules. The colors refer to the log_2_(fold changes), where red indicates up-regulation and green indicates down-regulation (see color key). The dendrograms that were obtained from hierarchical cluster analysis of both samples and molecules are shown adjacent to the heatmap. The color bar on the right indicates the cluster membership of the samples.
(PDF)

Figure S10 **Common genes between our inferred and published signatures for C vs. NC discrimination.** The Venn diagram illustrates the common genes between our signature inferred from mRNA data for the discrimination of carcinogens and non-carcinogens and signatures for the same class contrast previously published by Ellinger-Ziegelbauer *et al.* and Nakayama *et al.*
(PDF)

Table S1 **Signatures for C vs. NC discrimination.** The tables list the signatures inferred for the discrimination of carcinogenic and non-carcinogenic chemicals for each signature type (single-platform features: mRNA, miRNA, and protein expression as well as cross-platform features: molecular interaction (MI) and pathway enrichment (PE) features).
(XLS)

Table S2 **Signatures for NGC vs. GC discrimination.** The tables list the signatures inferred for the discrimination of non-genotoxic and genotoxic carcinogens for each signature type (single-platform features: mRNA, miRNA, and protein expression as well as cross-platform features: molecular interaction (MI) and pathway enrichment (PE) features).
(XLS)

Table S3 **Signatures for NGC vs. NC discrimination.** The tables list the signatures inferred for the discrimination of non-genotoxic carcinogens and non-carcinogens for each signature type (single-platform features: mRNA, miRNA, and protein expression as well as cross-platform features: molecular interaction (MI) and pathway enrichment (PE) features).
(XLS)
