# Supplementary material for: Cross-Platform Toxicogenomics for the Prediction of Non-Genotoxic Hepatocarcinogenesis in Rat
Source: PLoS One. 2014 May 15;9(5):e97640. doi: 10.1371/journal.pone.0097640 (PMC4022579; doi:10.1371/journal.pone.0097640)
Supplement: Figure S1 — Number of selected principle components for PCR. The histograms show the distribution of the optimal number of principal components selected during the parameter optimization for PCR. Parameter optimization is performed unbiased and independent of the estimation of classification performance for all relevant parameters of the individual classification algorithms. In our experiments, we performed 10 repetitions with different cross-validation splits for each repetition. In each repetition, a 2×2 cross-validation was used, leading to a total of 20 sets of optimized parameters. For PCR, the number of principal components is the only relevant parameter. The distribution of the 20 optimal numbers of principal components for each combination of features is shown in the histograms. (PDF) [file pone.0097640.s001.pdf]

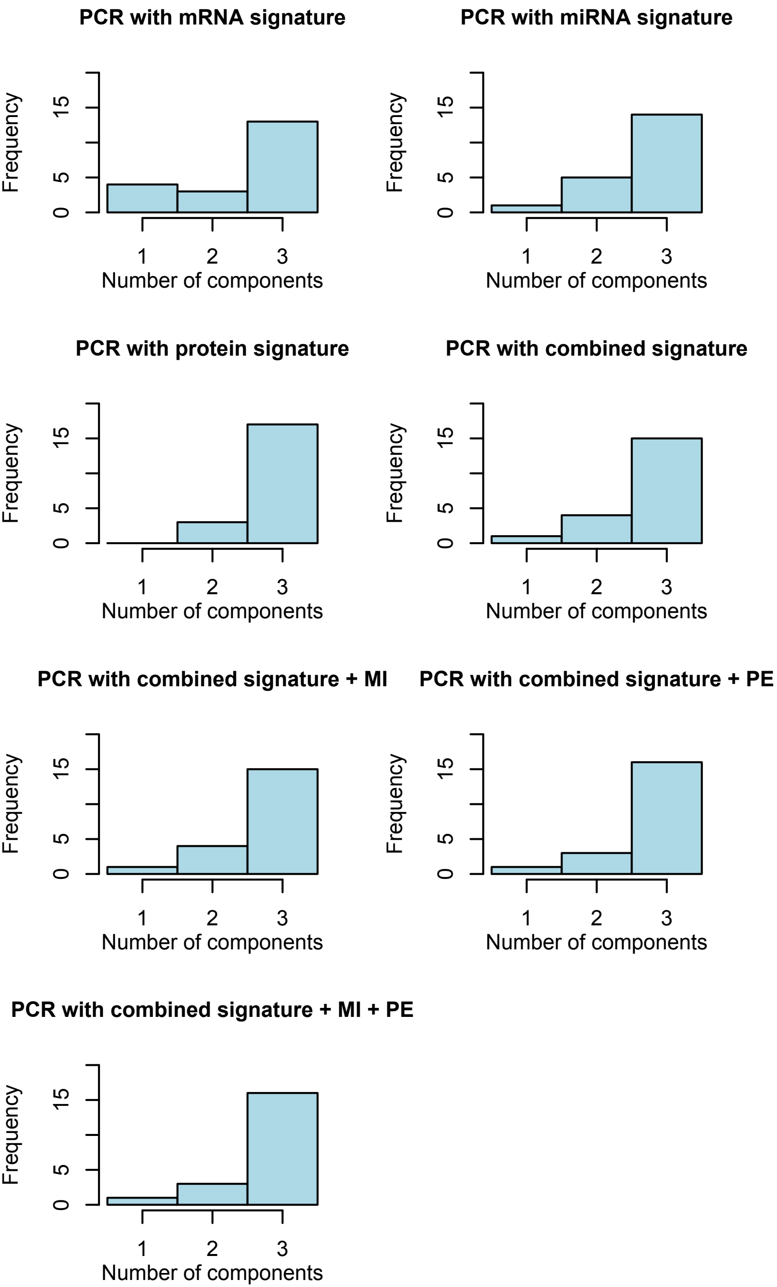
PCR with mRNA signature
PCR with miRNA signature
PCR with protein signature
PCR with combined signature
PCR with combined signature + MI
PCR with combined signature + PE
PCR with combined signature + MI + PE
Frequency
0
5
15
1
2
3
Number of components
