# Supplementary material for: Cross-Platform Toxicogenomics for the Prediction of Non-Genotoxic Hepatocarcinogenesis in Rat
Source: PLoS One. 2014 May 15;9(5):e97640. doi: 10.1371/journal.pone.0097640 (PMC4022579; doi:10.1371/journal.pone.0097640)
Supplement: Figure S2 — Cross-validation performance of signatures for compound classification. (A) The receiver operating characteristic (ROC) curves illustrate the predictive power of the signatures inferred for discrimination of carcinogens (C) and non-carcinogens (NC). The left plot illustrates the predictive power of the signature inferred using only mRNA features. The right plot illustrates the predictive power of the signature combining the single-platform signatures for mRNA, miRNA, and protein features as well as the cross-platform molecular interaction (MI) and pathway enrichment (PE) features. For each supervised learning method trained on the extracted signature, one ROC curve has been plotted. The dotted line indicates the chance level, which corresponds to an area under the ROC curve (AUC) of 0.5. The bar plot inside the ROC curve plot indicates the AUC achieved by each classifier. Predictive power was assessed using a 10 times repeated, nested 2×2-fold cross-validation. Prediction scores were scaled linearly to [0,1] and subsequently merged across all folds and repetitions to obtain a single ROC curve. (B) Predictive power of signatures for discrimination of non-genotoxic carcinogens (NGC) and genotoxic carcinogens (GC) illustrated as in (A). (C) Predictive power of signatures for discrimination of NGCs and NCs illustrated as in (A). (PDF) [file pone.0097640.s002.pdf]

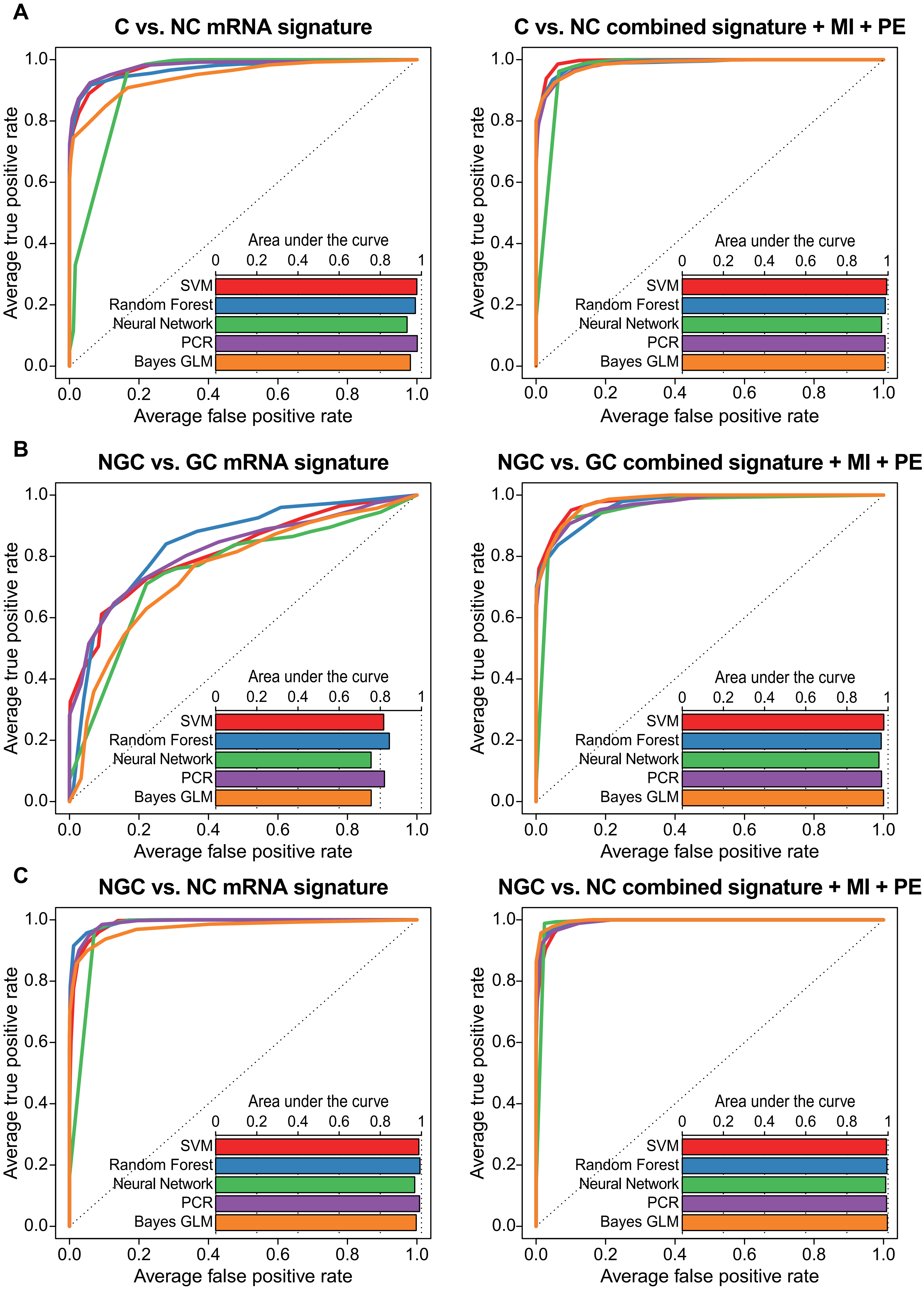
A
C vs. NC mRNA signature
C vs. NC combined signature + MI + PE
B
NGC vs. GC mRNA signature
NGC vs. GC combined signature + MI + PE
C
NGC vs. NC mRNA signature
NGC vs. NC combined signature + MI + PE
Average true positive rate
Average false positive rate
0.0
0.2
0.4
0.6
0.8
1.0
Area under the curve
0
0.2
0.4
0.6
0.8
1
SVM
Random Forest
Neural Network
PCR
Bayes GLM
