# Supplementary figures and images for: Cross-Platform Toxicogenomics for the Prediction of Non-Genotoxic Hepatocarcinogenesis in Rat
Source: PLoS One. 2014 May 15;9(5):e97640. doi: 10.1371/journal.pone.0097640 (PMC4022579; doi:10.1371/journal.pone.0097640)

A

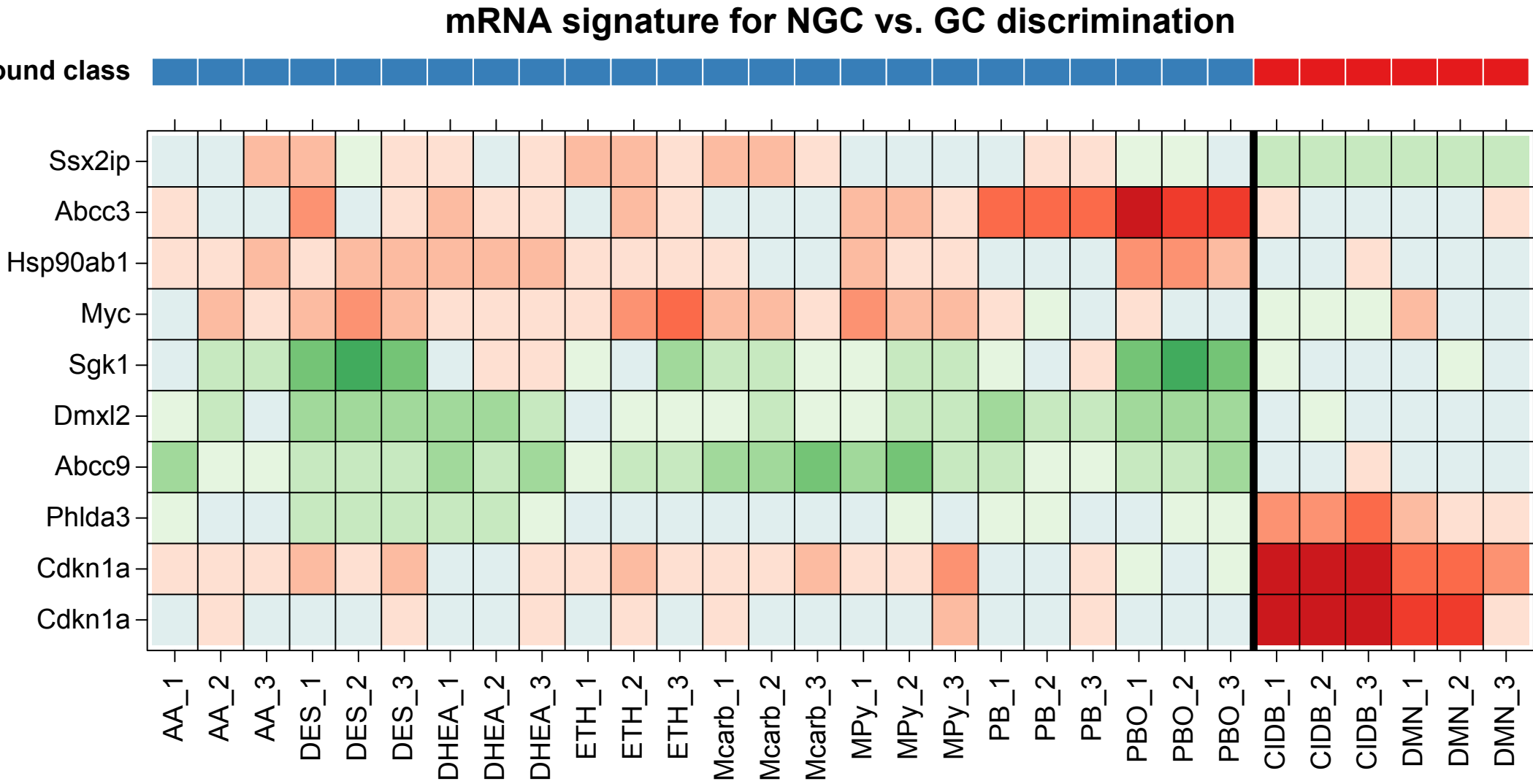

B

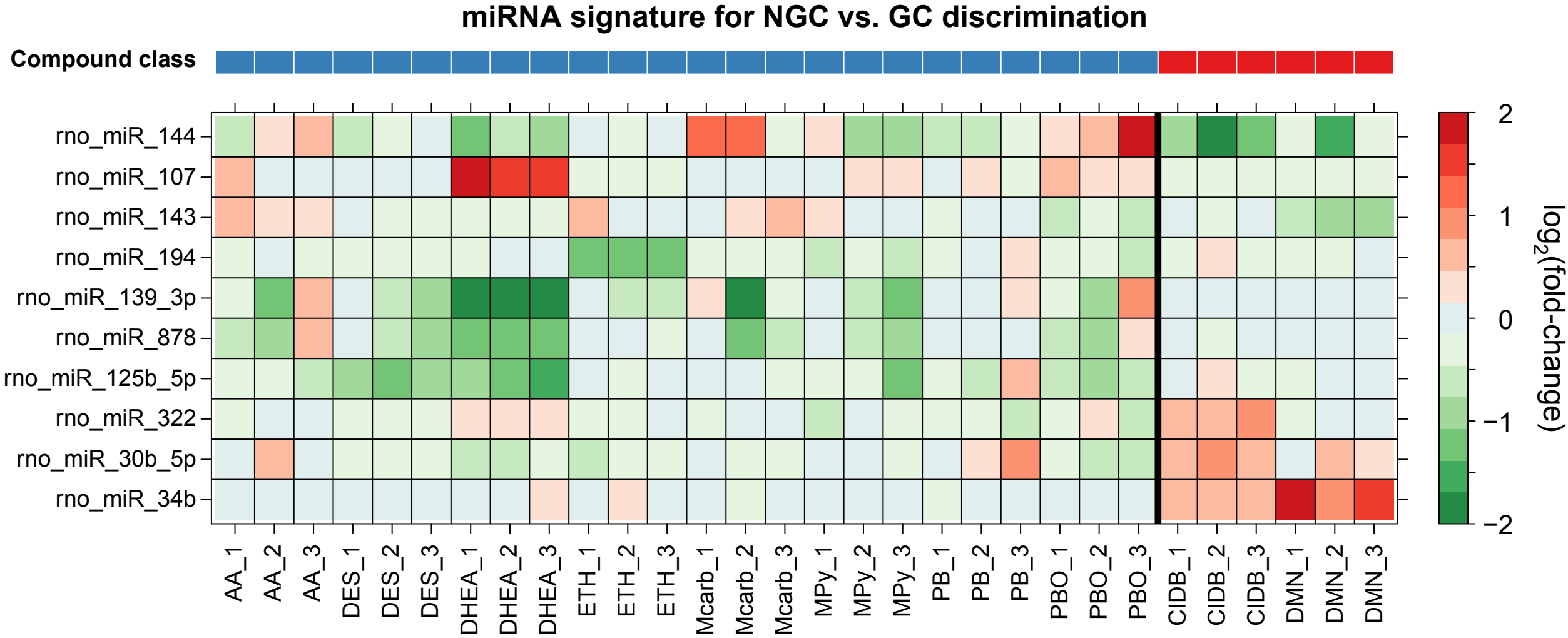

C

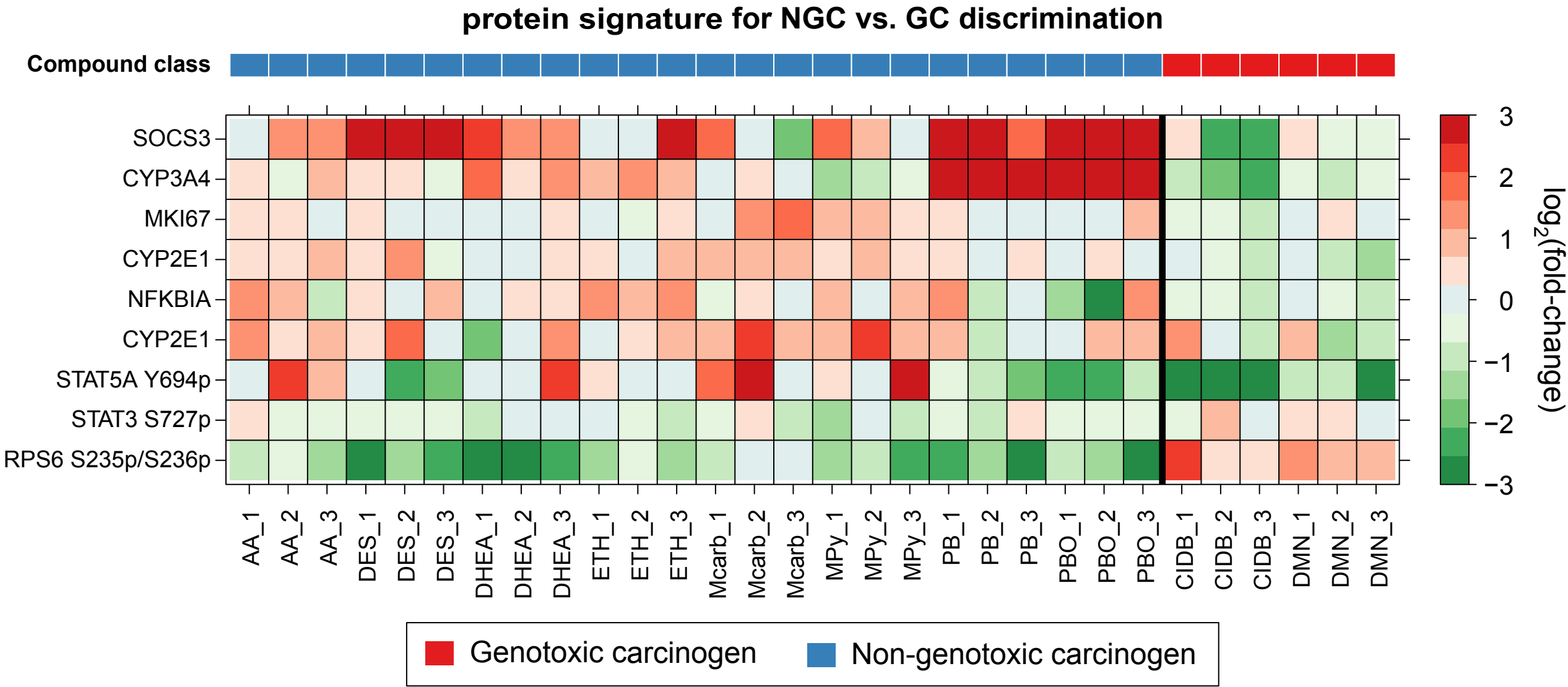

Supplement: Figure S3 — Heatmap plots of single-platform signatures for NGC vs. GC classification. The heatmaps depict characteristic expression patterns that were observed in livers of rats after exposure to non-genotoxic or genotoxic rodent hepatocarcinogens. A selection of signature molecules is shown for each profiled molecular level: (A) mRNA expression, (B) miRNA expression, and (C) protein expression. In each heatmap, the rows correspond to signature molecules and the columns correspond to differentially liver samples from differentially treated rats. The bold vertical lines separate the NGCs from the GCs. Plotted are the log2(fold changes), where red indicates up-regulation and green indicates down-regulation (see color keys). The color bar on top refers to the carcinogenic compound class (see legend). (PDF) [file pone.0097640.s003.pdf]

**AA (NGC)**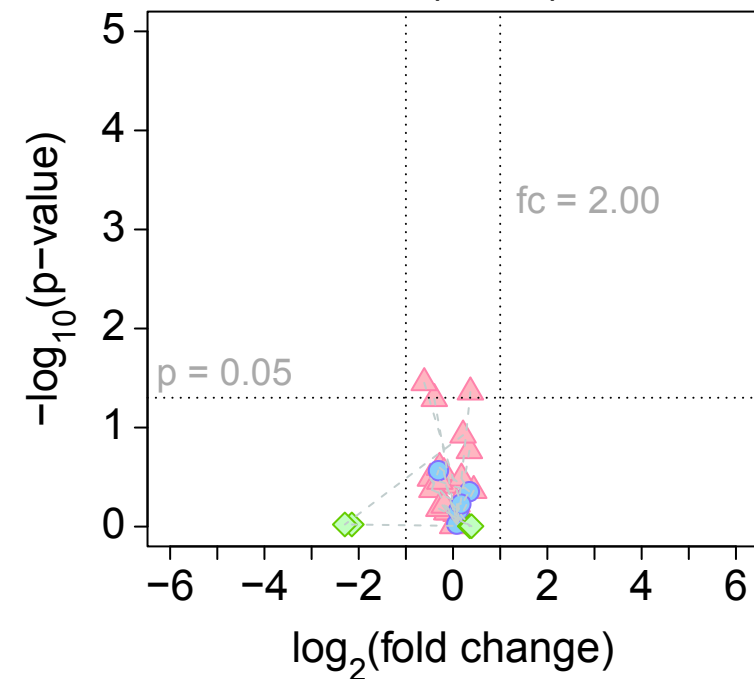**DHEA (NGC)**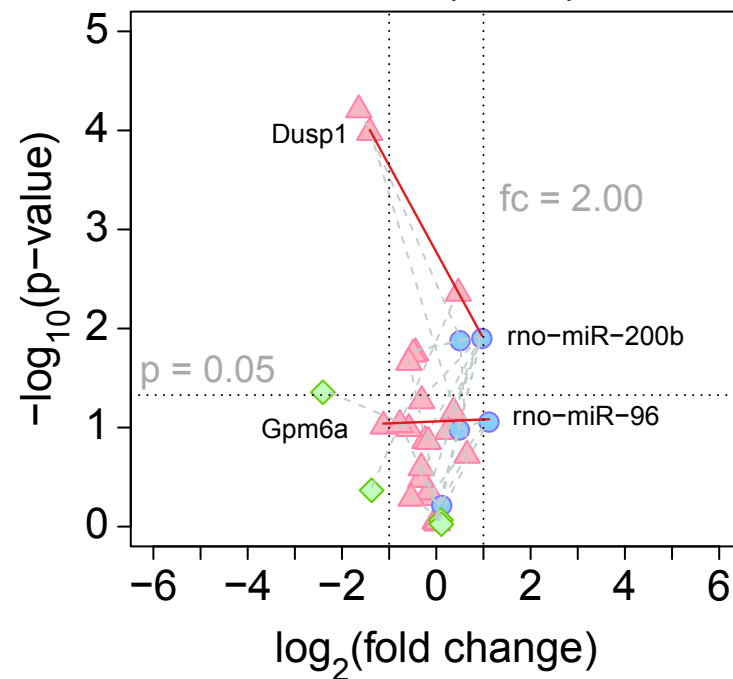**ETH (NGC)**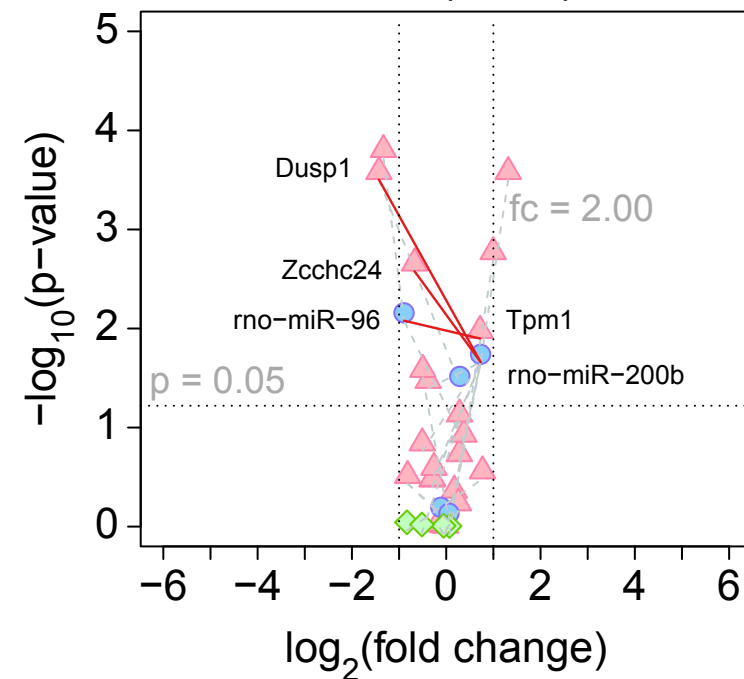**Mcarb (NGC)**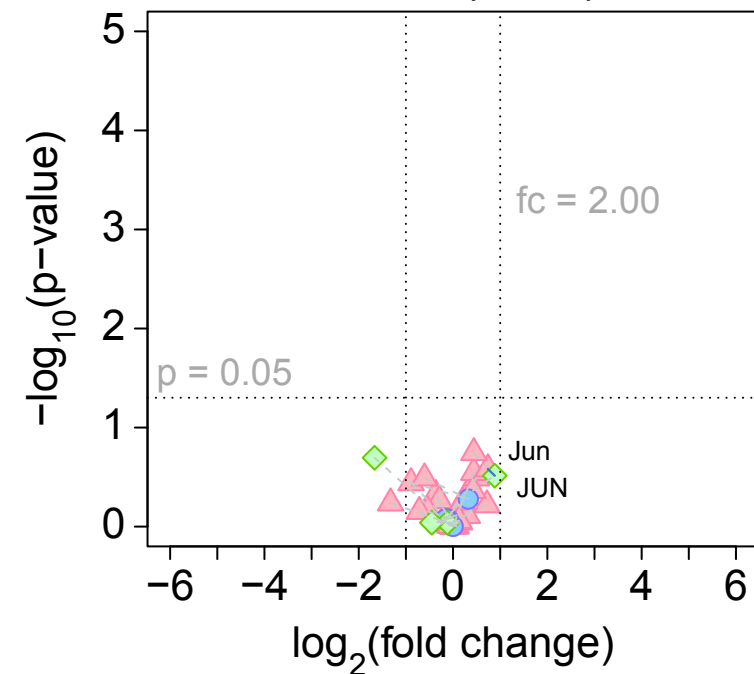**MPy (NGC)**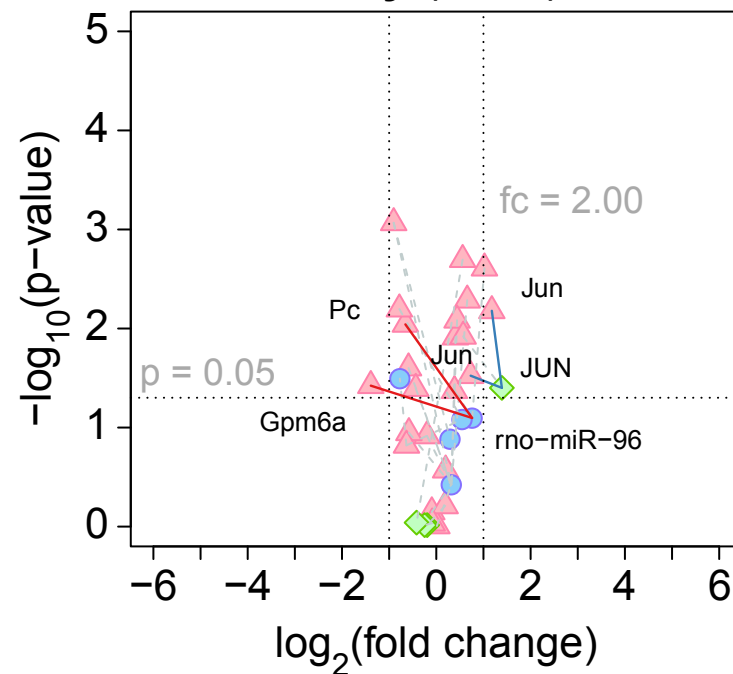**PB (NGC)**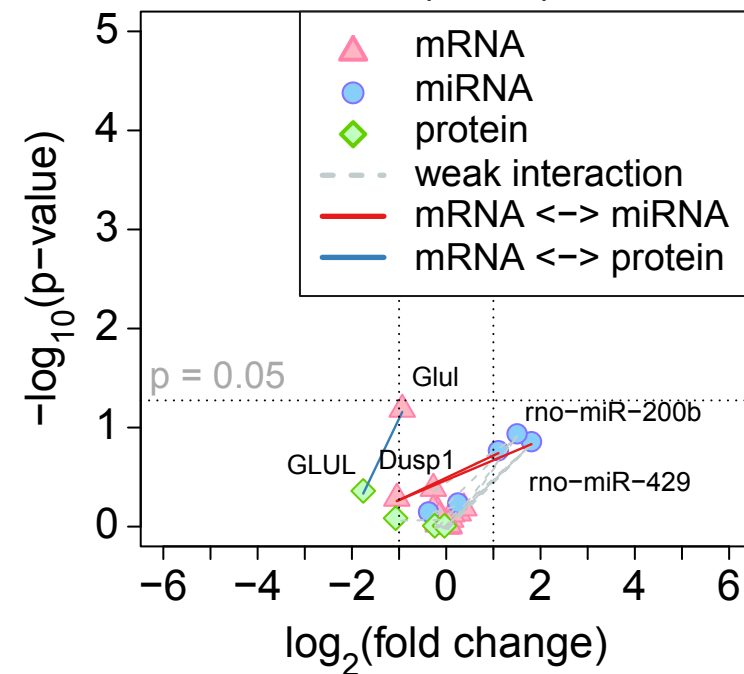

Supplement: Figure S6 — Volcano plots of molecular interaction signatures. Shown are volcano plots for expression profiles of the six non-genotoxic carcinogens not shown in Figure 6. The plots represent putative molecular interactions between different molecular layers, which were found to be predictive for C vs. NC classification. For each interacting molecule (i.e., mRNA, miRNA, or protein), the strength of its differential expression was assessed in terms of the log2(fold change) and plotted against its significance which is given by the FDR-corrected log10(p-value) obtained from a moderated t-test. Different shapes and colors denote different types of molecules (see legend). Colored edges were used to highlight molecular interactions for which a positive or negative correlation was observed between two platforms. We considered correlations in the expression profiles of miRNAs and their experimentally confirmed or predicted mRNA targets as well as between mRNAs and proteins sharing the same genomic locus. As a formal criterion for a putative molecular interaction, we required that for both interaction partners a 50% increase or decrease in expression was observed relative to the controls. (PDF) [file pone.0097640.s006.pdf]

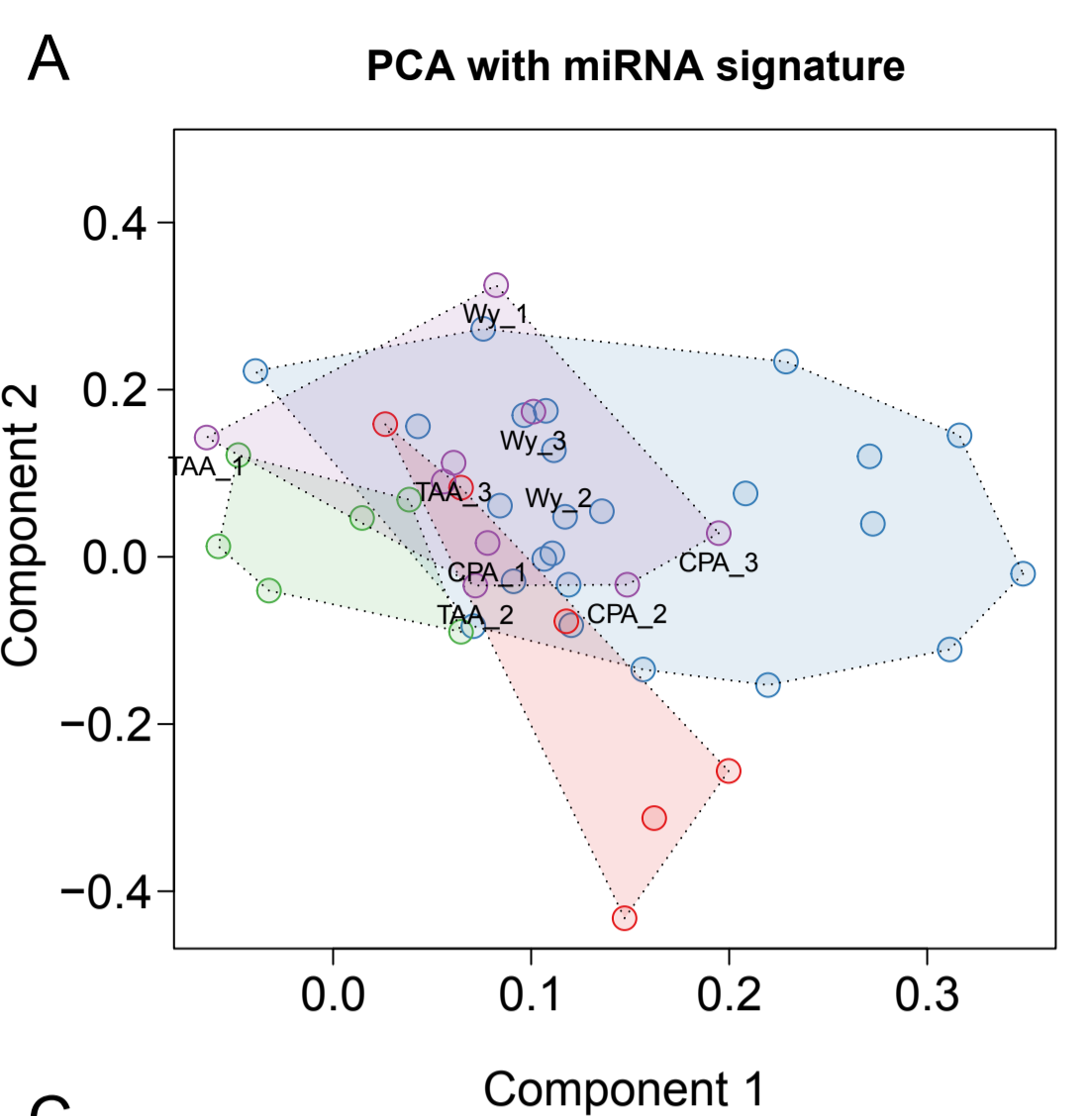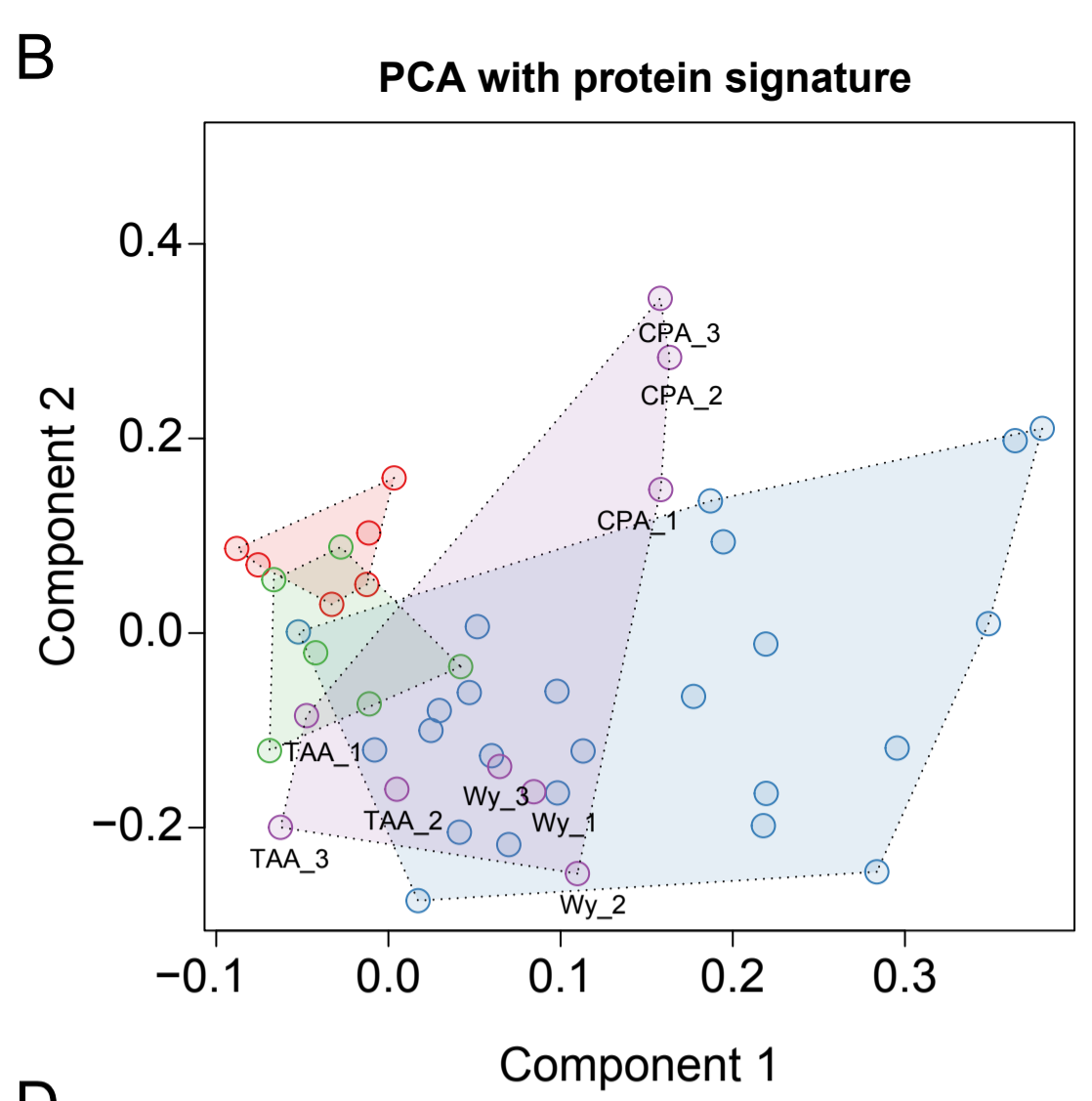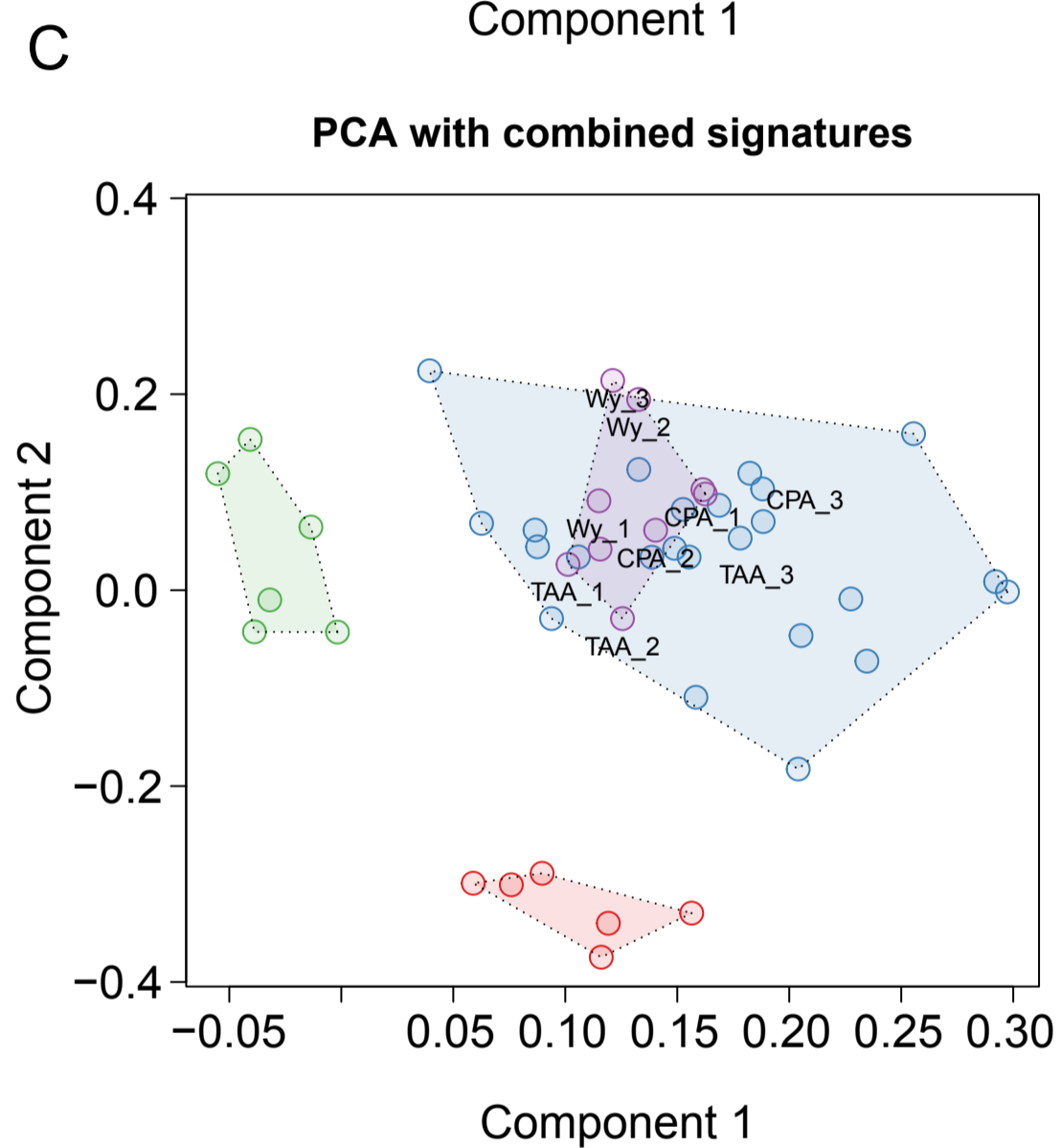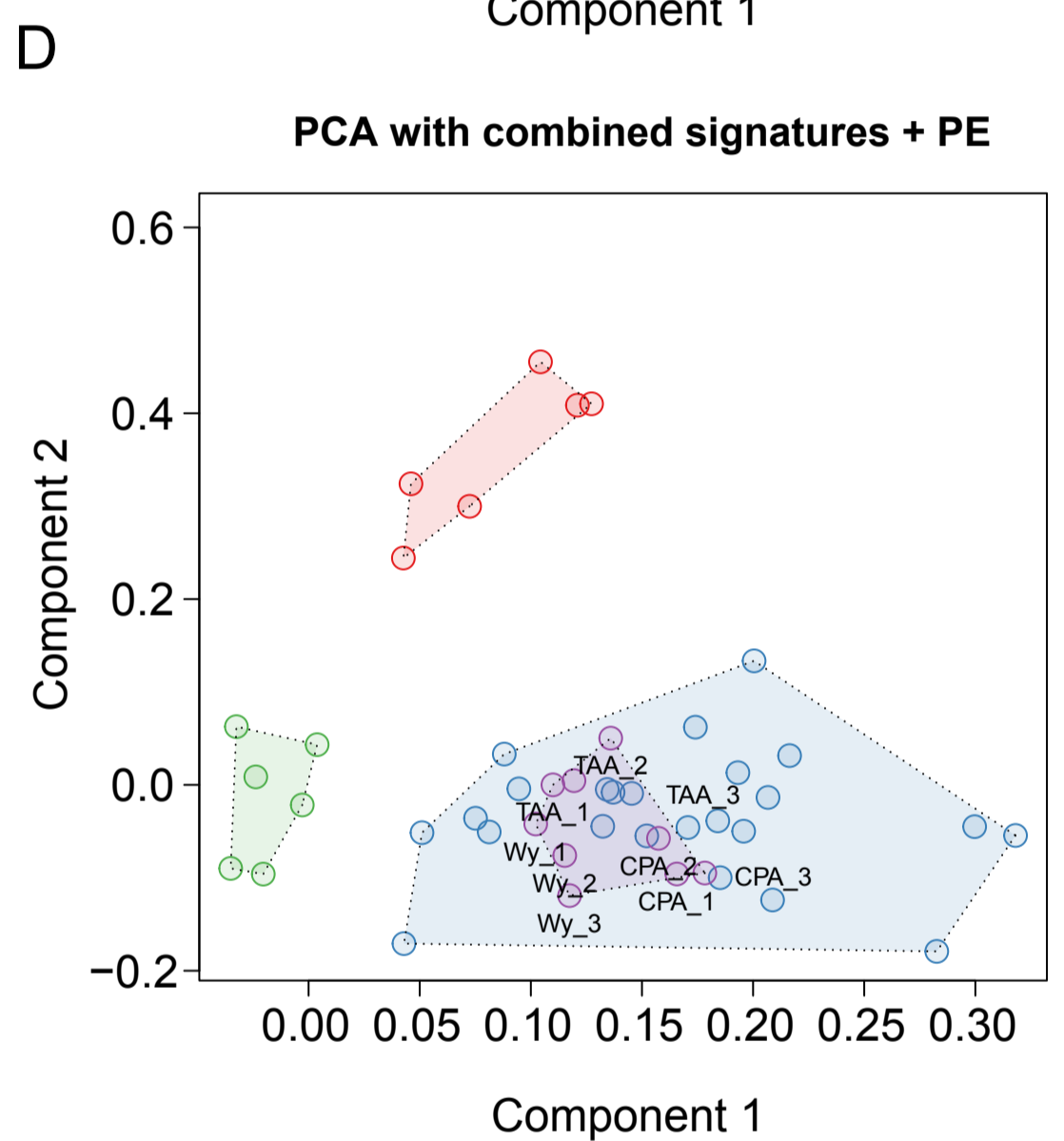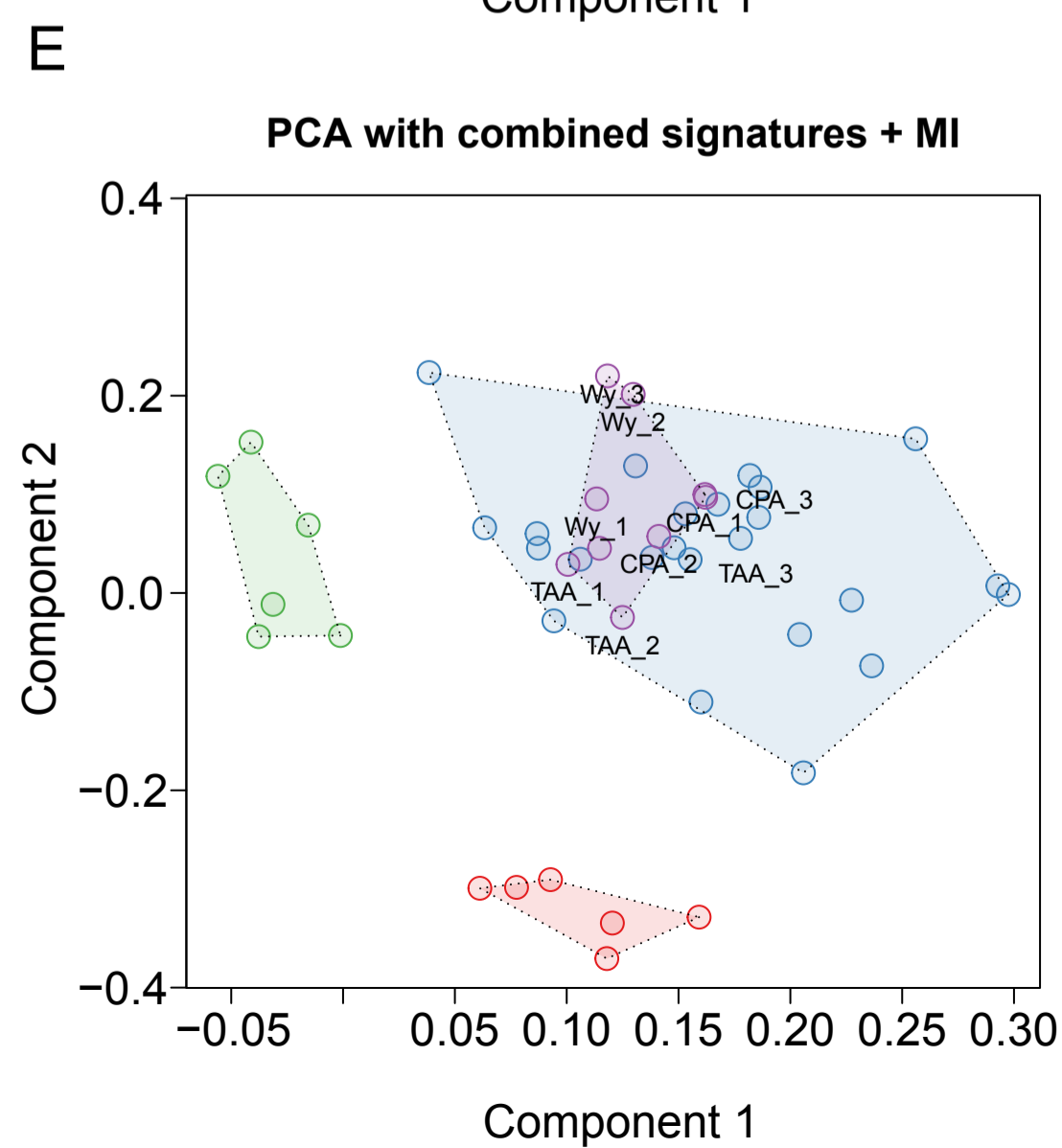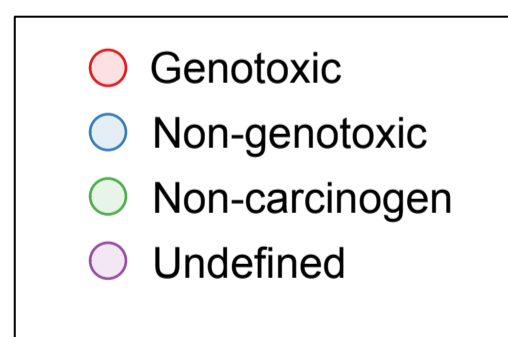

Supplement: Figure S7 — Principal component analysis of signatures for classification of undefined compounds. (A) Samples are represented based on the mRNA signature for NGC vs. GC discrimination. The corresponding fold changes were PCA-transformed and plotted in a lower-dimensional space spanned by the first two principal components. The color of the spheres corresponds to the class of the administered compounds with respect to their hepatocarcinogenic properties in rats. Clusters of rat liver samples after treatment of rats with compounds of the same class are highlighted by means of transparent polygons. (B) Similar plot as in (A), but based on the miRNA signature for NGC vs. GC discrimination. (C) Similar plot as in (A), but instead of using a single-platform signature for sample representation, all single-platform (mRNA, miRNA, protein) signatures were combined. (D) Similar plot as in (A), but instead of using a single-platform signature for sample representation, all single-platform (mRNA, miRNA, protein) signatures and the cross-platform pathway enrichment (PE) signature were combined. (E) Similar plot as in (A), but instead of using a single-platform signature for sample representation, all single-platform (mRNA, miRNA, protein) signatures and the cross-platform molecular interaction (MI) signature were combined. (PDF) [file pone.0097640.s007.pdf]

Ellinger-Ziegelbauer et al.

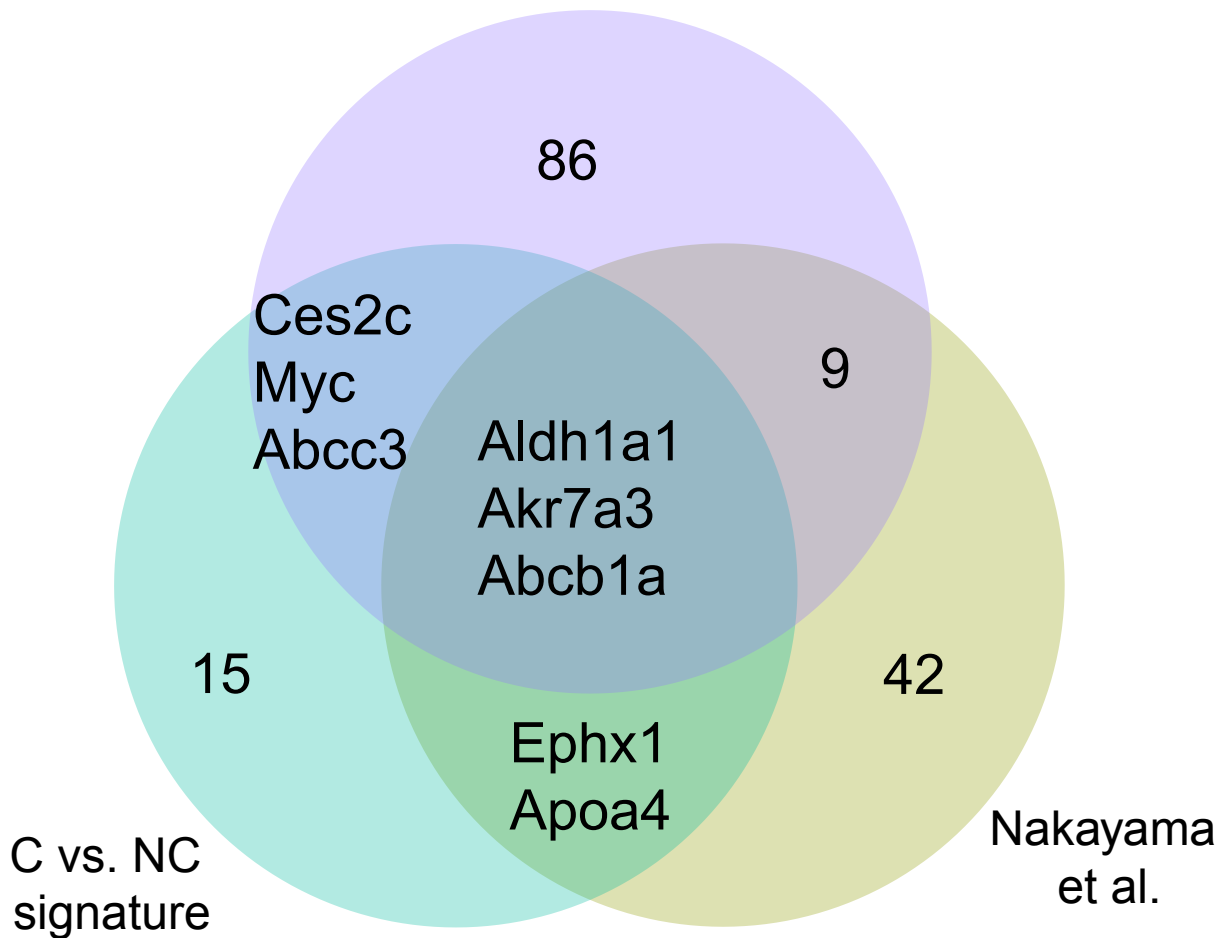

Supplement: Figure S10 — Common genes between our inferred and published signatures for C vs. NC discrimination. The Venn diagram illustrates the common genes between our signature inferred from mRNA data for the discrimination of carcinogens and non-carcinogens and signatures for the same class contrast previously published by Ellinger-Ziegelbauer et al. and Nakayama et al. (PDF) [file pone.0097640.s010.pdf]
