# Supplementary material for: Cross-Platform Toxicogenomics for the Prediction of Non-Genotoxic Hepatocarcinogenesis in Rat
Source: PLoS One. 2014 May 15;9(5):e97640. doi: 10.1371/journal.pone.0097640 (PMC4022579; doi:10.1371/journal.pone.0097640)
Supplement: Figure S4 — Heatmap plots of single-platform signatures for NGC vs. NC classification. The heatmaps depict characteristic expression patterns, which were observed in livers of rats after exposure to non-genotoxic hepatocarcinogens and non-carcinogens. A selection of signature molecules is shown for each profiled molecular level: (A) mRNA expression, (B) miRNA expression, and (C) protein expression. In each heatmap, the rows correspond to signature molecules and the columns correspond to differentially treated rat liver samples. The bold vertical lines separate the NGCs from the NCs. Plotted are the log2(fold changes), where red indicates up-regulation and green indicates down-regulation (see color keys). The color bar on top refers to the carcinogenic compound class (see legend). (PDF) [file pone.0097640.s004.pdf]

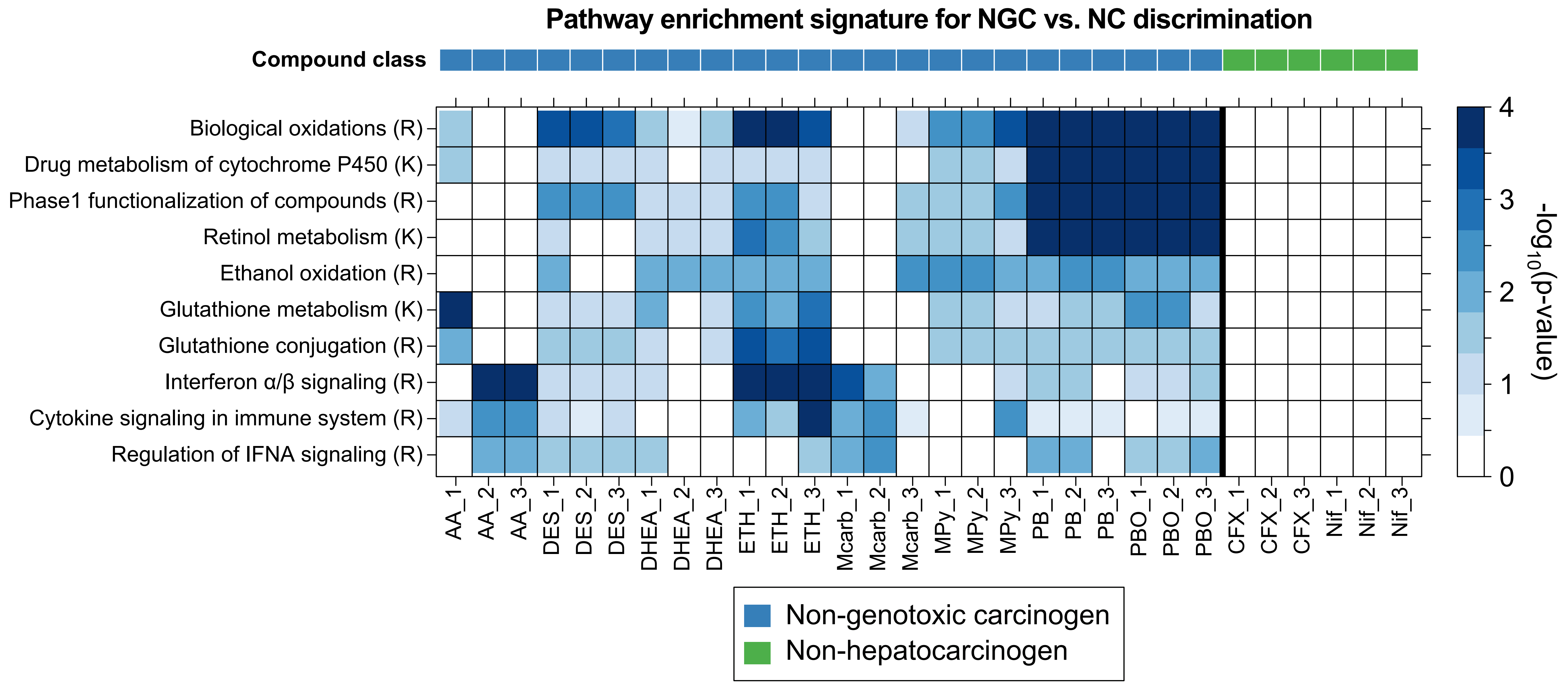

Pathway enrichment signature for NGC vs. NC discrimination
Compound class
Biological oxidations (R)
Drug metabolism of cytochrome P450 (K)
Phase1 functionalization of compounds (R)
Retinol metabolism (K)
Ethanol oxidation (R)
Glutathione metabolism (K)
Glutathione conjugation (R)
Interferon α/β signaling (R)
Cytokine signaling in immune system (R)
Regulation of IFNA signaling (R)
AA_1 AA_2 AA_3 DES_1 DES_2 DES_3 DHEA_1 DHEA_2 DHEA_3 ETH_1 ETH_2 ETH_3 Mcarb_1 Mcarb_2 Mcarb_3 MPy_1 MPy_2 MPy_3 PB_1 PB_2 PB_3 PBO_1 PBO_2 PBO_3 CFX_1 CFX_2 CFX_3 Nif_1 Nif_2 Nif_3
-log10(p-value)
0 1 2 3 4
Non-genotoxic carcinogen
Non-hepatocarcinogen
