# Supplementary material for: Cross-Platform Toxicogenomics for the Prediction of Non-Genotoxic Hepatocarcinogenesis in Rat
Source: PLoS One. 2014 May 15;9(5):e97640. doi: 10.1371/journal.pone.0097640 (PMC4022579; doi:10.1371/journal.pone.0097640)
Supplement: Figure S5 — Heatmap plot for pathway enrichment. The heatmap depicts overrepresentation of genes involved in relevant pathways among the genes deregulated upon treatment with a certain compound. Pathways relevant for compound classification were selected by SVM-RFE for NGC vs. NC discrimination. The rows correspond to canonical pathways from the databases Reactome (R), KEGG (K), or BioCarta (B) and the columns correspond to samples. The color of each cell refers to the -log10(p-value) obtained from a hypergeometric overrepresentation test and indicates the significance of a certain pathway enrichment (see color key). The color bar on top of each heatmap denotes the carcinogenic class (see legend). (PDF) [file pone.0097640.s005.pdf]

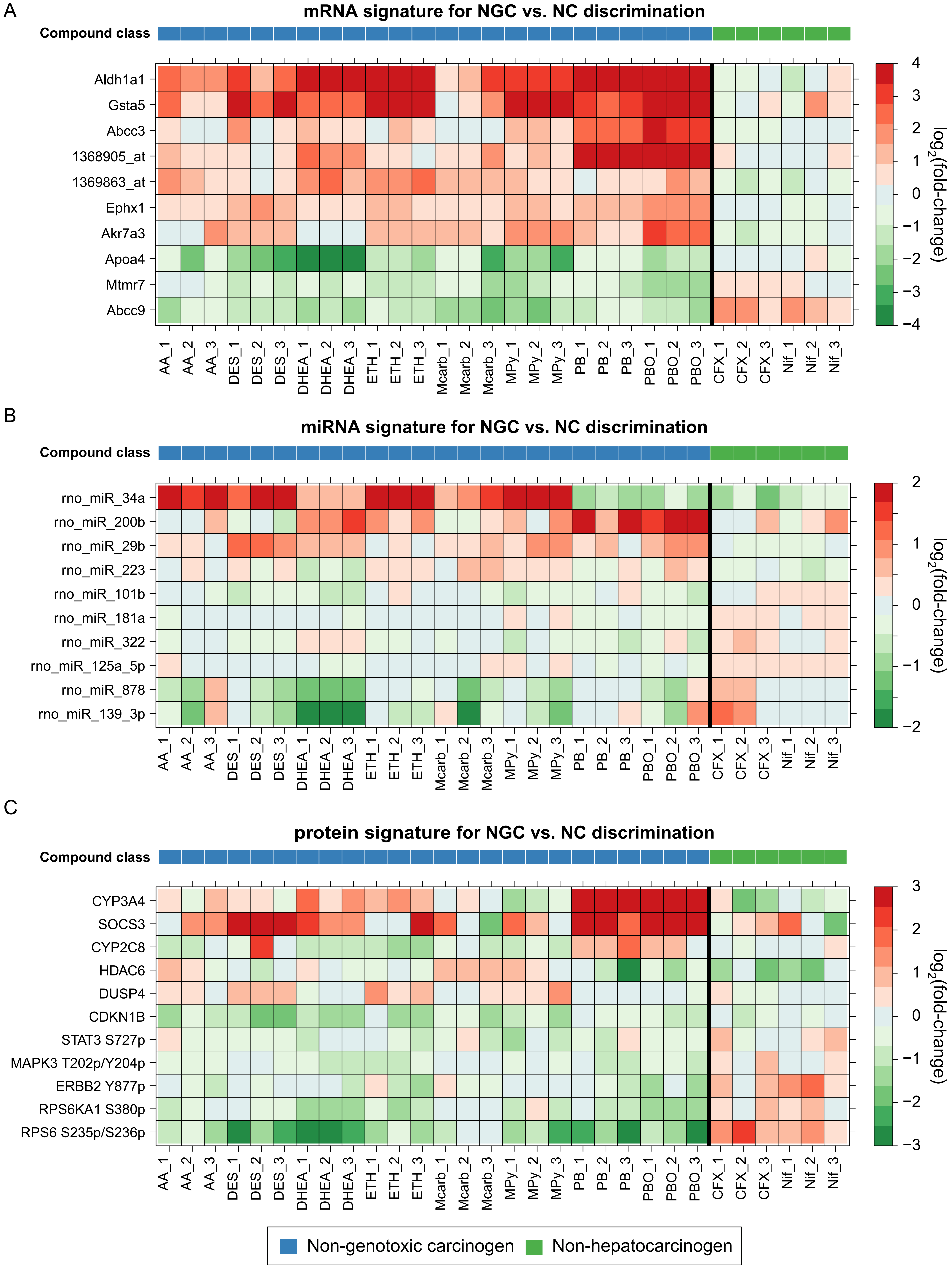
A
mRNA signature for NGC vs. NC discrimination
Compound class
Aldh1a1
Gsta5
Abcc3
1368905_at
1369863_at
Ephx1
Akr7a3
Apoa4
Mtmr7
Abcc9
AA_1 AA_2 AA_3 DES_1 DES_2 DES_3 DHEA_1 DHEA_2 DHEA_3 ETH_1 ETH_2 ETH_3 Mcarb_1 Mcarb_2 Mcarb_3 MPy_1 MPy_2 MPy_3 PB_1 PB_2 PB_3 PBO_1 PBO_2 PBO_3 CFX_1 CFX_2 CFX_3 Nif_1 Nif_2 Nif_3
4 3 2 1 0 −1 −2 −3 −4
$\log_2$(fold-change)
B
miRNA signature for NGC vs. NC discrimination
Compound class
rno_miR_34a
rno_miR_200b
rno_miR_29b
rno_miR_223
rno_miR_101b
rno_miR_181a
rno_miR_322
rno_miR_125a_5p
rno_miR_878
rno_miR_139_3p
AA_1 AA_2 AA_3 DES_1 DES_2 DES_3 DHEA_1 DHEA_2 DHEA_3 ETH_1 ETH_2 ETH_3 Mcarb_1 Mcarb_2 Mcarb_3 MPy_1 MPy_2 MPy_3 PB_1 PB_2 PB_3 PBO_1 PBO_2 PBO_3 CFX_1 CFX_2 CFX_3 Nif_1 Nif_2 Nif_3
2 1 0 −1 −2
$\log_2$(fold-change)
C
protein signature for NGC vs. NC discrimination
Compound class
CYP3A4
SOCS3
CYP2C8
HDAC6
DUSP4
CDKN1B
STAT3 S727p
MAPK3 T202p/Y204p
ERBB2 Y877p
RPS6KA1 S380p
RPS6 S235p/S236p
AA_1 AA_2 AA_3 DES_1 DES_2 DES_3 DHEA_1 DHEA_2 DHEA_3 ETH_1 ETH_2 ETH_3 Mcarb_1 Mcarb_2 Mcarb_3 MPy_1 MPy_2 MPy_3 PB_1 PB_2 PB_3 PBO_1 PBO_2 PBO_3 CFX_1 CFX_2 CFX_3 Nif_1 Nif_2 Nif_3
3 2 1 0 −1 −2 −3
$\log_2$(fold-change)
Non-genotoxic carcinogen
Non-hepatocarcinogen
