# Supplementary material for: Cross-Platform Toxicogenomics for the Prediction of Non-Genotoxic Hepatocarcinogenesis in Rat
Source: PLoS One. 2014 May 15;9(5):e97640. doi: 10.1371/journal.pone.0097640 (PMC4022579; doi:10.1371/journal.pone.0097640)
Supplement: Figure S8 — Reclassification of undefined compounds. (A) The heatmap displays the confidence scores obtained from five different machine learning methods that were used to classify the undefined compounds CPA, TAA, and WY as either NGC or GC based on the mRNA signature for NGC vs. GC discrimination. The confidence scores are [0, 1]-scaled and correspond to the probability that a certain sample was derived from rats treated with an NGC (see color key). (B) Similar illustration as in (A) obtained from an SVM classifier trained on the miRNA signature. (C) Similar illustration as in (A) obtained from an SVM classifier trained on all single-platform (mRNA, miRNA, protein) signatures combined. (D) Similar illustration as in (A) obtained from an SVM classifier trained on all single-platform (mRNA, miRNA, protein) signatures and the cross-platform pathway-enrichment (PE) signature combined. (E) Similar illustration as in (A) obtained from an SVM classifier trained on all single-platform (mRNA, miRNA, protein) signatures and the cross-platform molecular interaction (MI) signature combined. (PDF) [file pone.0097640.s008.pdf]

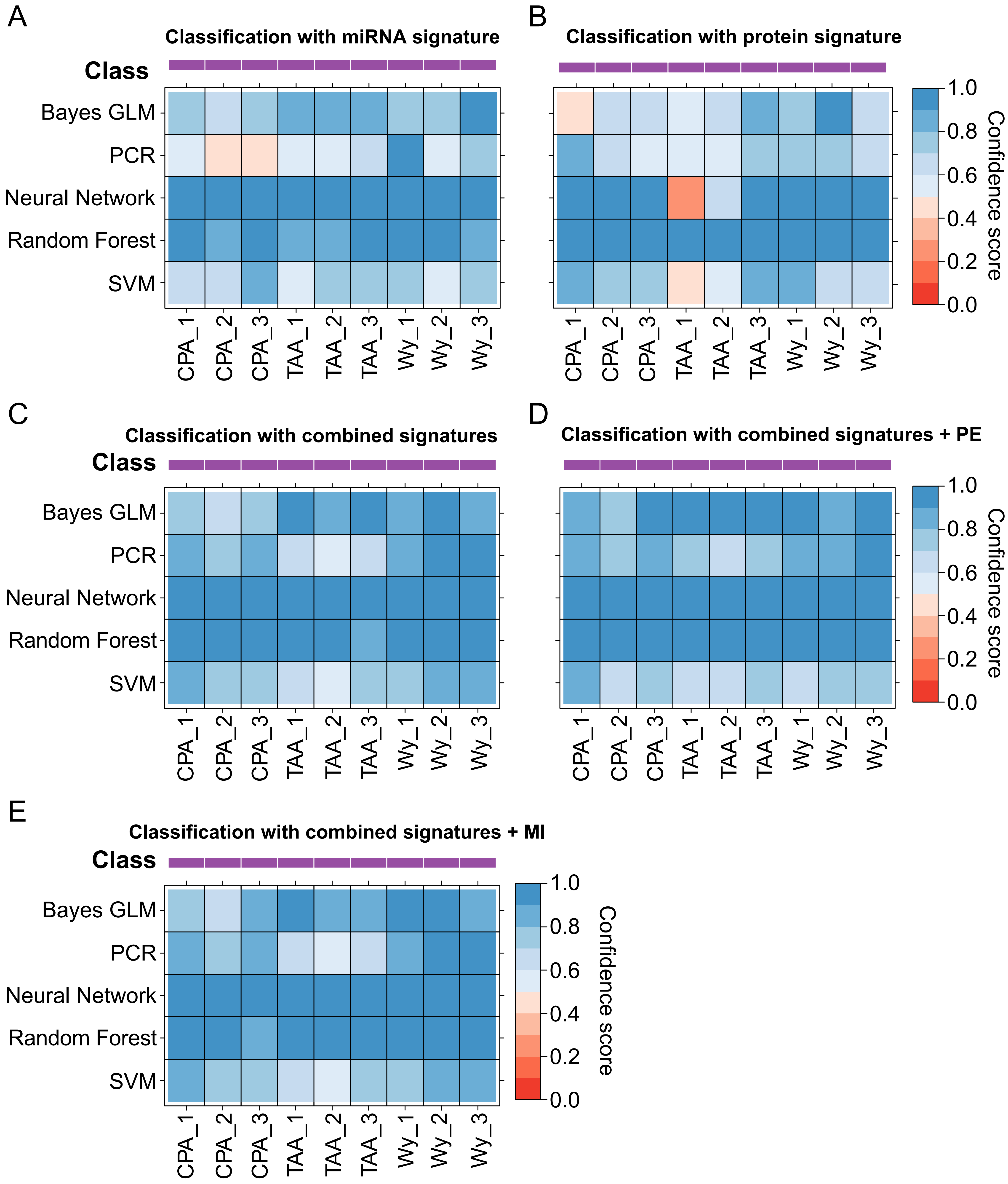
A
Classification with miRNA signature
Class
Bayes GLM
PCR
Neural Network
Random Forest
SVM
CPA_1
CPA_2
CPA_3
TAA_1
TAA_2
TAA_3
Wy_1
Wy_2
Wy_3
B
Classification with protein signature
1.0
0.8
0.6
0.4
0.2
0.0
Confidence score
C
Classification with combined signatures
D
Classification with combined signatures + PE
E
Classification with combined signatures + MI
