# Supplementary material for: Cross-Platform Toxicogenomics for the Prediction of Non-Genotoxic Hepatocarcinogenesis in Rat
Source: PLoS One. 2014 May 15;9(5):e97640. doi: 10.1371/journal.pone.0097640 (PMC4022579; doi:10.1371/journal.pone.0097640)
Supplement: Figure S9 — Cluster analysis of mRNA, miRNA, and protein expression data. The heatmaps depict the results of a cluster analysis limited to the top 100 differentially expressed (A) mRNAs, (B) miRNAs, and (C) proteins in livers of rats treated with diverse carcinogenic or non-carcinogenic compounds. In each heatmap, the rows correspond to the animals and the columns correspond to the profiled molecules. The colors refer to the log2(fold changes), where red indicates up-regulation and green indicates down-regulation (see color key). The dendrograms that were obtained from hierarchical cluster analysis of both samples and molecules are shown adjacent to the heatmap. The color bar on the right indicates the cluster membership of the samples. (PDF) [file pone.0097640.s009.pdf]

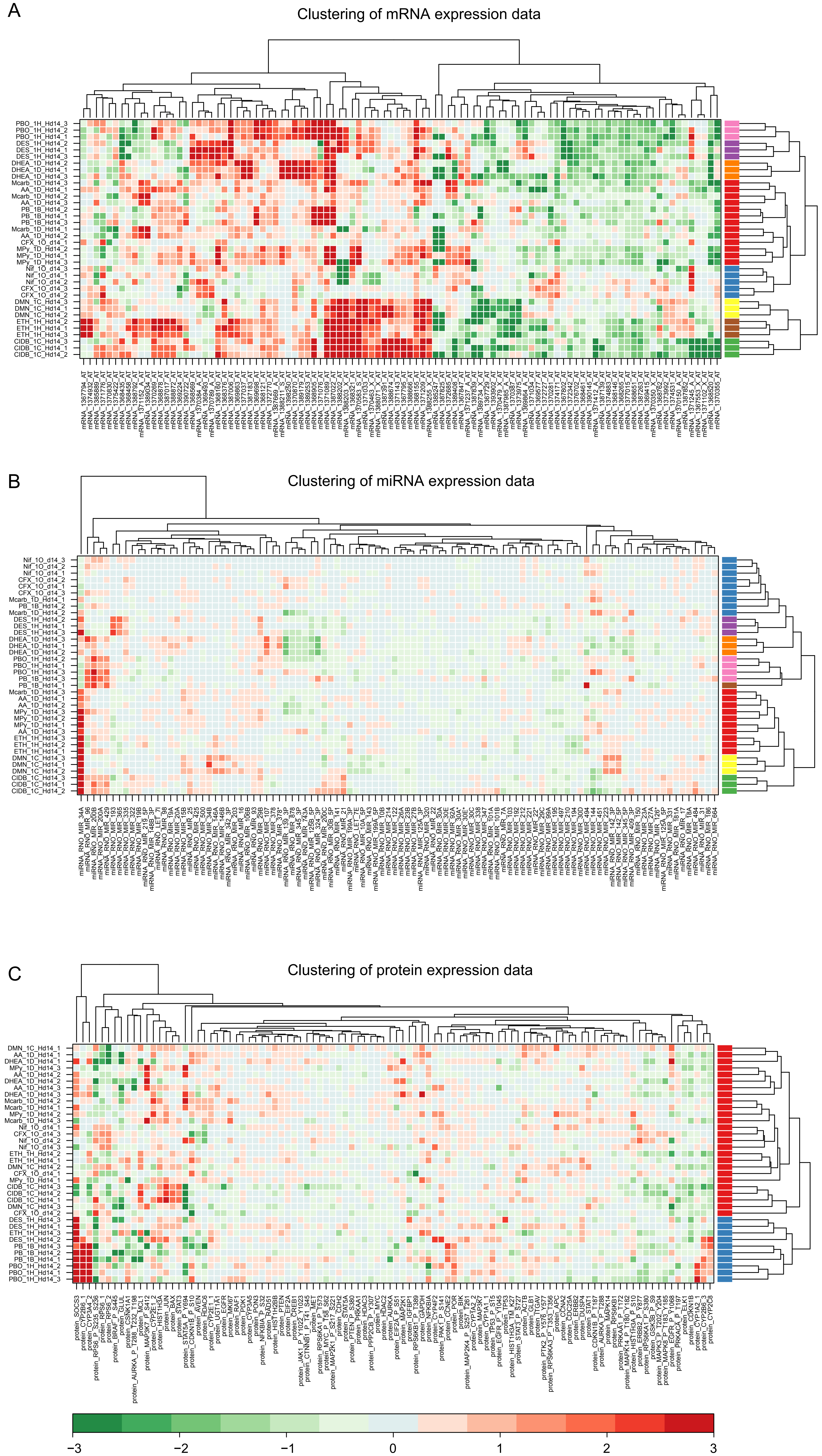
A
Clustering of mRNA expression data
B
Clustering of miRNA expression data
C
Clustering of protein expression data
−3
−2
−1
0
1
2
3
